# Updated global prevalence and ethnic diversity of von Willebrand disease based on population genetics analysis

**DOI:** 10.1038/s41598-026-36145-6

**Published:** 2026-01-20

**Authors:** Omid Seidizadeh, Andrea Cairo, Camilla Oriani, Flora Peyvandi

**Affiliations:** 1 Department of Pathophysiology and Transplantation, Università degli Studi di Milano, Milan, Italy; 2 Angelo Bianchi Bonomi Hemophilia and Thrombosis Center, Fondazione IRCCS Ca’Granda Ospedale Maggiore Policlinico, Milan, Italy

**Keywords:** VWD prevalence, gnomAD, Genetic prevalence, VWF gene, Genome, Genomics, Medical genetics, Genetics, Medical research, Molecular medicine, Diseases, Haematological diseases

## Abstract

von Willebrand disease (VWD) is known as the most common inherited bleeding disorder. Despite this, some uncertainty remains regarding its prevalence and the distribution of different phenotypes worldwide and across ethnic groups. By leveraging genetic epidemiology approaches that enable population-scale estimates, the prevalence of VWD and its diverse phenotypes can be assessed more precisely. We estimated the global VWD prevalence and its distribution across distinct ethnic groups using the largest and most widely used publicly available genetic database. Available exome/genome sequencing data of 807,162 (730,947 exomes, 76,215 genomes) subjects from the Genome Aggregation Database (gnomAD-v4.1) were used. Based on the allele frequencies of 321 previously reported *VWF* pathogenic variants identified in the gnomAD population, the estimated worldwide prevalence of VWD per 1,000 individuals was 10.6 for type 1, 1.3 for type 2A, 1.7 for type 2B, and 1.5 for type 2 M. For the recessive forms of VWD, the global prevalence was estimated to be 33.9 cases per million for type 2N and 1.3 cases per million for type 3 VWD. Once structural variants and copy number variants from gnomAD that result in loss of function were included, the estimated prevalence of type 3 slightly increased to 1.8 per million. Additionally, VWD prevalence was found to vary significantly across different ethnic groups. This updated population-based genetic prevalence indicates a substantially higher than expected frequency of VWD. Our novel finding suggests that a large number of patients remain undiagnosed.

**Supplementary Information:**

The online version contains supplementary material available at 10.1038/s41598-026-36145-6.

## Introduction

Von Willebrand factor (VWF), primarily synthesized in endothelial cells and megakaryocytes, is a large glycoprotein that plays a crucial role in hemostasis^1^. VWF is essential for the early stages of primary hemostasis, because it facilitates platelet adhesion to damaged blood vessel walls by binding to both platelets and subendothelial collagen. In addition, VWF serves as the carrier for clotting factor VIII, protecting it from degradation and ensuring its availability at sites of vascular injury^1^. von Willebrand disease (VWD) is the most common inherited bleeding disorder, caused by genetic variants in the VWF gene (*VWF*), resulting in a deficiency or dysfunction of VWF^1,2^. The quantitative defect of VWF can be partial or complete leading to type 1 or type 3 VWD. Qualitative defects result in four different types 2 (2A, 2B, 2M, and 2N)^2,3^. The inheritance pattern of VWD is both autosomal dominant (type 1, 2A, 2B, and 2M) and recessive (type 2N and type 3) forms^4^.

Previous studies estimated the VWD prevalence to range from 0.6% to 1.3%^5,6^. However, based on cases referred to specialized treatment centers, the prevalence of clinically relevant VWD is estimated to be approximately 1 in 1,000 individuals^7^. The true prevalence of VWD is likely to be underestimated, because many patients remain asymptomatic until they encounter hemostatic challenges. In addition, underestimation can be attributed to limited clinician awareness of the disease, the complexity of diagnostic testing, and frequent misdiagnosis^8^. Furthermore, the sample sizes in the three aforementioned studies were insufficient to accurately estimate the global prevalence of VWD, as they were limited to a small number of geographic regions. This sample size limitation further prevented the inclusion of all six VWD types, thus hampered the estimation of the true global prevalence as well as the prevalence of each phenotype.

With the advent of large-scale and population-based sequencing studies, genetic prevalence—the proportion of a population carrying a causal genotype for a genetic disorder—has become an increasingly valuable tool for estimating the number of affected globally individuals and in various populations^9^. We previously estimated the prevalence of VWD using the genome Aggregation Database (gnomAD v2), which includes more than 141,000 individual’s exome and genome sequences^10^. The gnomAD v4.1 has been recently released with nearly 5x larger sample size than the combined gnomAD v2^9^ and gnomAD v3^11^ releases by including sequencing data of 807,162 subjects.

In this study, we aimed to estimate the global and within-population prevalence of VWD by utilizing gnomAD v4.1, i.e., the largest and most widely used publicly available genetic database. Additionally, we aimed to estimate the prevalence of each VWD type. All analyses were performed using variants that had been thoroughly evaluated based on American College of Medical Genetics and Genomics/ Association for Molecular Pathology (ACMG/AMP) guidelines, ClinVar classifications, and in silico prediction tools.

## Results

### Global mutational spectrum of the VWF gene

High-quality sequencing data of *VWF* was collected from the gnomAD population of 807,162 subjects (1,614,324 alleles) with different ethnicities (Table 1). The population includes African/African American (*n* = 37,545), Admixed American (*n* = 30,019), Ashkenazi Jewish (*n* = 14,804), East Asian (*n* = 22,448), Finnish (*n* = 32,026), Middle Eastern (*n* = 3,031), non-Finnish European (*n* = 590,031), South Asian (*n* = 45,546), and Remaining, with no assigned ethnicity (*n* = 31,712). The average depth of sequencing coverage per base in all *VWF* exons (except exon 26) was almost always greater than 30x for exome and genome sequencing, (Supplementary Fig. S1), indicating adequate coverage of the gene. The lower coverage of exon 26 is primarily due to alignment of the sequences with human genome reference, being aligned with the pseudogene instead of the *VWF.*^12^ Since gnomAD sets a minimum depth of coverage at 10 (DP ≥ 10), only genotypes meeting this threshold were included in this study; exon 26 having a coverage depth exceeding this threshold. Among the 1,614,324 alleles analyzed in the gnomAD population, 10,397 distinct variants were identified in the *VWF*.

**Table 1:** The gnomAD population stratified by ethnicity.

Population	Total
All	807,162
African/African American	37,545
Admixed American	30,019
Ashkenazi Jewish	14,804
East Asian	22,448
Finnish	32,026
Middle Eastern	3,031
European (non-Finnish)	590,031
South Asian	45,546
Remaining	31,712

### Selected pathogenic variants

Due to next generation sequencing (NGS) limitations in detecting large or complex variants, our analysis primarily focused on 4,154 (among 10,397 distinct variants) SNVs and short insertions/deletions associated with VWD, including missense, splicing, stop-gain/loss, and small insertions/deletions. Exclusively for type 3 VWD, we performed an additional analysis including structural variants (SVs, *n* = 7) and copy number variants (CNVs, *n* = 9) reported in the gnomAD population that resulted in loss of function (*n* = 16) but not previously reported. Among these 4,154 variants, we excluded variants without a clear link to VWD and those classified as Benign or Likely benign in ClinVar (see Methods), resulting in 331 variants with MAFs < 0.01 considered potentially VWD-causing.

**Fig. 1:**
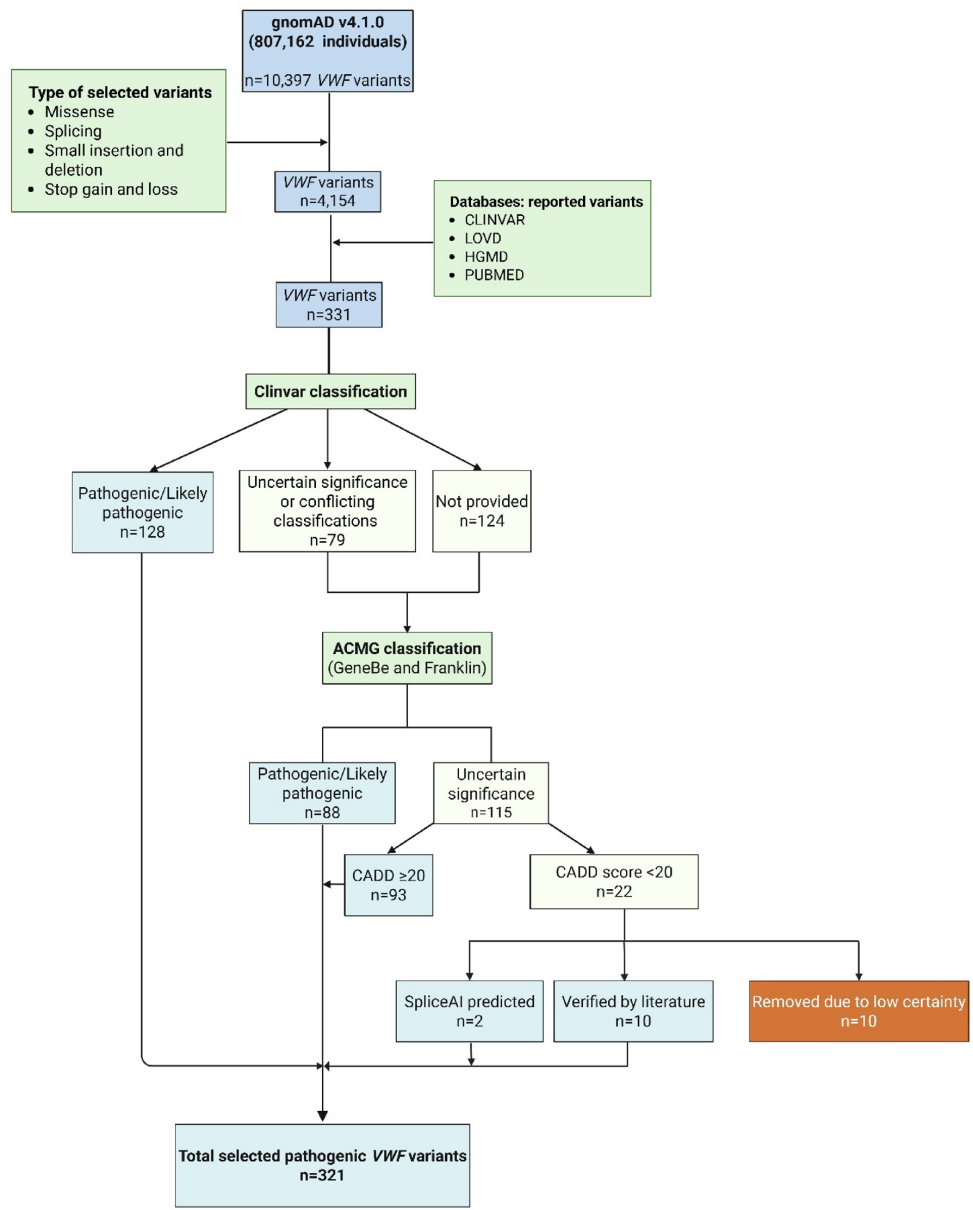
Flowchart of the data analysis pipeline used to identify previously reported pathogenic variants causing von Willebrand disease (VWD).

To refine this list, we applied further filtering (Fig. 1). ClinVar classified 128 variants as Pathogenic/Likely pathogenic, 79 as of uncertain significance or with conflicting interpretations, and 124 were unreported in ClinVar. The latter two groups underwent ACMG classification using GeneBe and Franklin tools, identifying 88 additional Pathogenic/Likely pathogenic variants and 115 of uncertain significance. Based on CADD scores, of the 115 variants, 93 had scores ≥ 20 (predicted pathogenic), while 22 were < 20. Of these 22 variants, 12 were retained based on additional evidence: 2 predicted to affect splicing by SpliceAI and 10 validated as VWD-causing in original studies. Ten variants were excluded due to low-confidence literature data. In total, 321 (SNVs and short insertions/deletions) variants were included for estimating global and population-specific VWD prevalence (Supplementary Table S1).

**Fig. 2:**
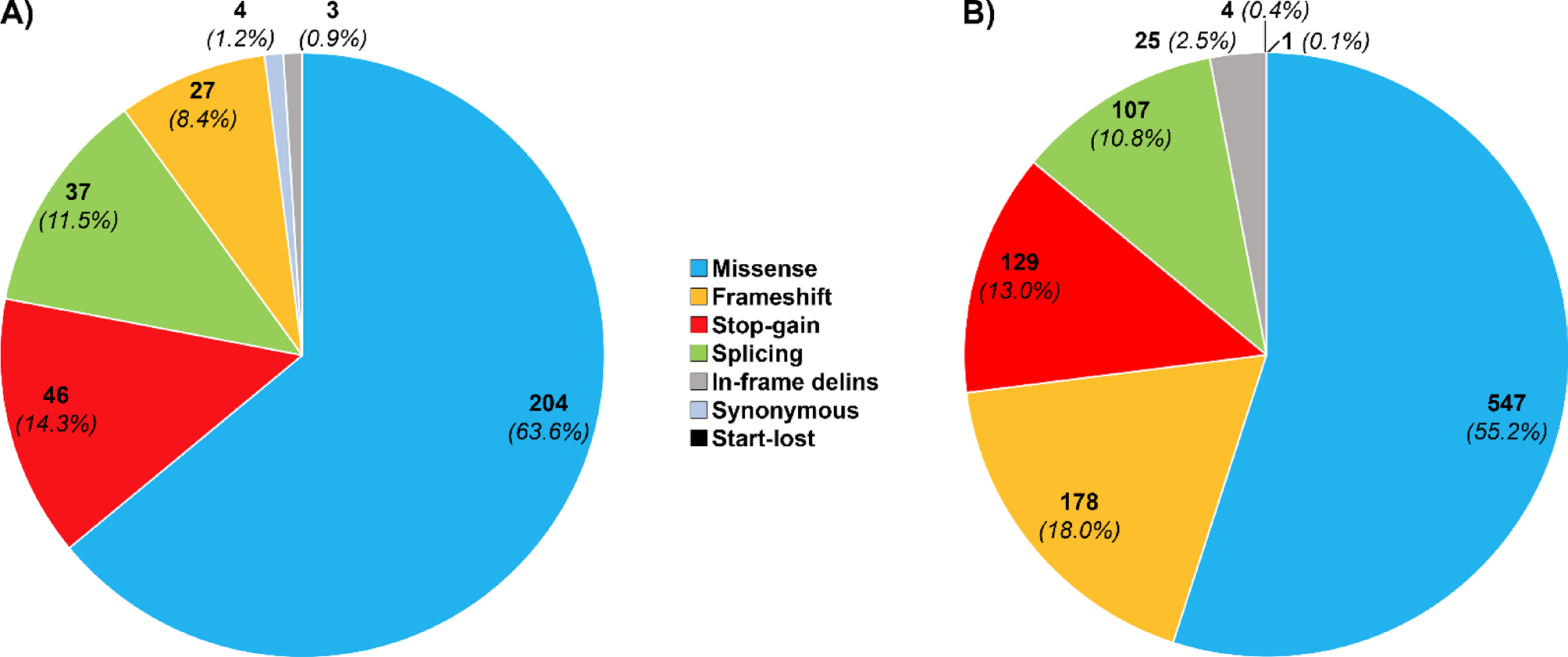
Distribution of mutation types for previously reported pathogenic variants identified in gnomAD and those reported so far to cause von Willebrand disease (VWD), including single nucleotide variants and short insertions/deletions. Panel (A) shows 321 distinct variants in gnomAD that have been previously reported and being classified as pathogenic/likely pathogenic, while panel (B) illustrates 991 variants that have been reported in total to cause VWD.

The distribution of mutation types for the 321 variants identified in the gnomAD v4.1 is depicted in Fig. 2A. Missense accounted for the majority of variants (*n* = 204, 63.6%) followed by stop-gained (*n* = 46, 14.3%). Variants affecting a splicing site (*n* = 37, 11.5%), frameshift (*n* = 27, 8.4%), synonymous (*n* = 4, 1.2%) as well as inframe delins (*n* = 3, 0.9%) were also identified.

### Mutational spectrum of all previously reported variants in association with VWD

Among SNVs and short insertion/deletion variants, 991 different variants were found to be associated with VWD in genetic databases (HGMD, LOVD, or ClinVar) or in the literature. Worth noting, we excluded all variants with no clear association with VWD (i.e., those not disease-causing), as well as those classified as Benign or Likely benign in ClinVar to reach these 991 variants. The distribution of mutation types for all previously reported variants associated with VWD is illustrated in Fig. 2B.

### Population-based prevalence of VWD

We calculated the global and population-specific prevalence of VWD for both autosomal dominant and recessive forms. Types 1, 2A, 2B, and 2M are inherited in a dominant manner, while types 3 and 2N are recessively inherited^4^. We used the allele frequencies of 321 gnomAD variants that were associated with VWD phenotypes, based on well-defined criteria for determining their disease-causing nature (see Methods). Accordingly, the estimated global prevalence of type 1 was 10.6 (95% CI 10.4–10.8) cases per 1000 people. For autosomal dominant types 2, we estimated a global prevalence of 1.3 (95% CI 1.2–1.4) cases per 1000 for 2 , 1.7 (95% CI 1.6–1.8) cases per 1000 for 2B and 1.5 (95% CI 1.4–1.6) cases per 1000 for 2M. For the recessive VWD forms, we estimated a global prevalence of 33.9 (95% CI 32.5–35.2) cases per million for type 2N and 1.3 (95% CI 1.2–1.5) case per million for type 3 (Table 2). The details of the within-population prevalence of VWD subtypes are summarized in Supplementary Tables S2-S7.

Once SVs and CNVs from gnomAD that resulted in loss of function were included, the estimated prevalence of type 3 VWD slightly increased to 1.8 per million (95% CI 1.65–1.96) (Supplementary Table S8).

**Table 2:** Global and within-population prevalence of von Willebrand disease (VWD).

Population	Type 1 per 10^3^	2A per 10^3^	2B per 10^3^	2M per 10^3^	2N per 10^6^	Type 3 per 10^6^
All	10.6 (10.4–10.8)	1.3 (1.2–1.4)	1.7 (1.6–1.8)	1.5 (1.4–1.6)	33.9 (32.5–35.2)	1.3 (1.2–1.5)
African/African American	8.2 (7.3–9.1)	1.0 (0.7–1.4)	0.5 (0.3–0.8)	0.7 (0.5–1.0)	4.2 (3.0–5.7)	0.4 (0.2–0.7)
Admixed American	7.2 (6.3–8.2)	2.9 (2.3–3.6)	2.5 (1.9–3.0)	1.4 (1.0–1.9)	10.9 (8.1–14.2)	0.2 (0.08–0.4)
Ashkenazi Jewish	7.3 (6.0–8.7)	0.3 (0.07–0.5)	6.6 (5.3–7.9)	0.1 (0–0.2)	2.0 (1.0–3.4)	0.01 (0–0.06)
East Asian	4.4 (3.6–5.3)	4.4 (3.6–5.3)	0.04 (0–0.13)	1.0 (0.6–1.4)	0.1 (0.04–0.29)	0.4 (0.2–0.8)
European Finnish	8.2 (7.3–9.2)	1.7 (1.2–2.1)	8.9 (7.8–9.9)	0.03 (0–0.09)	44.7 (36.7–53.6)	1.3 (0.8–2.1)
Middle Eastern	10.5 (6.9–14.4)	2.3 (0.7–4.3)	1.0 (0–2.3)	3.0 (1.3–4.9)	2.2 (0.4–6.1)	0.7 (0.03–2.7)
European (non-Finnish)	12.0 (11.7–12.2)	1.1 (1.0–1.2)	1.3 (1.2–1.4)	1.0 (1.0–1.1)	46.2 (44.2–48.2)	1.0 (0.9–1.2)
South Asian	3.3 (2.8–3.8)	1.1 (0.8–1.4)	0.9 (0.7–1.2)	9.7 (8.8–10.6)	4.4 (3.2–5.8)	19.5 (16.0–23.6)
Remaining	9.7 (8.6–10.8)	2.1 (1.6–2.7)	1.9 (1.4–2.4)	2.2 (1.7–2.7)	27.9 (22.4–34.2)	1.3 (0.8–2.0)

Values in parentheses represent 95% confidence intervals (CIs).

### Most frequent pathogenic variants stratified by ethnicity

**Fig. 3:**
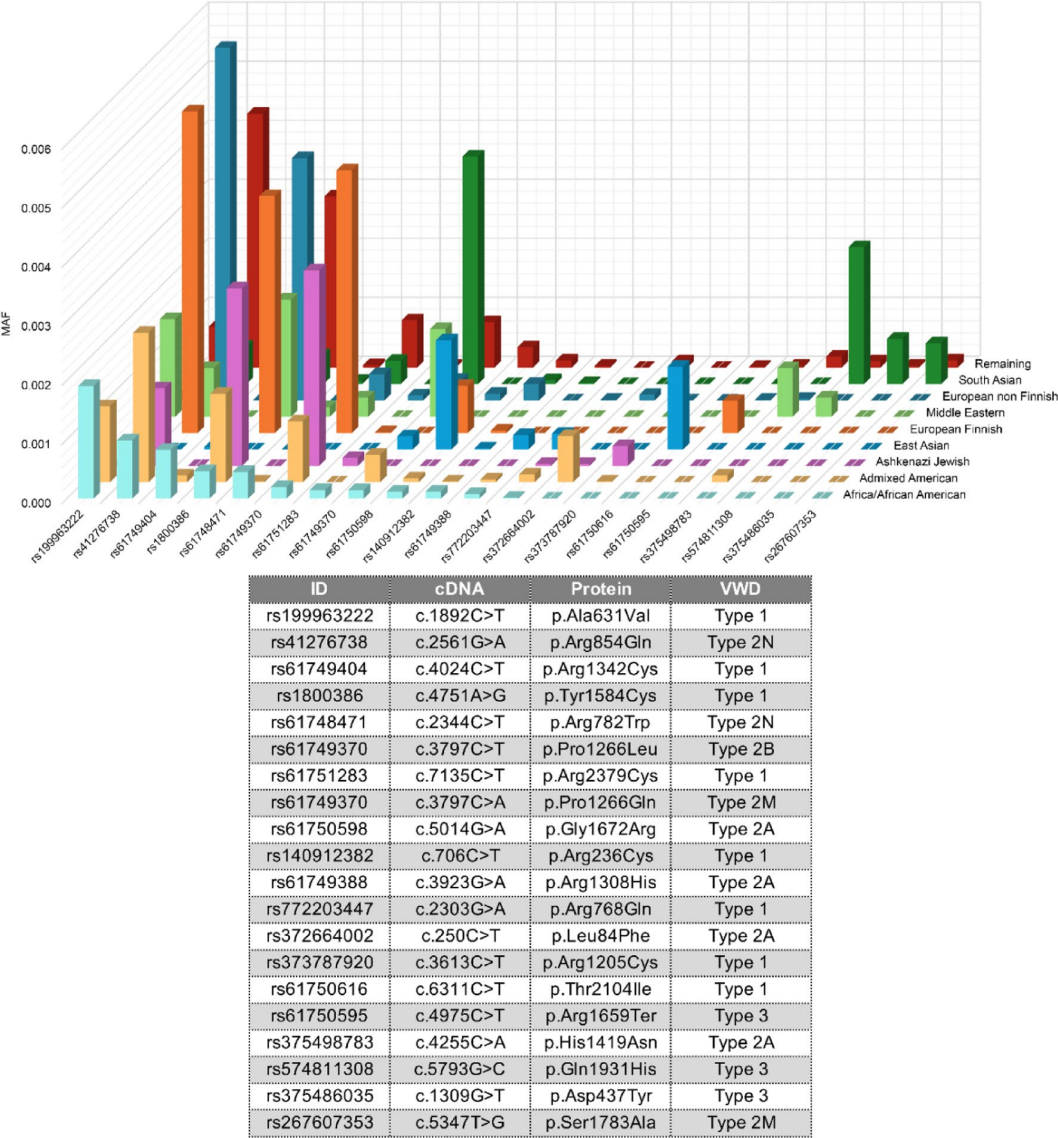
Ethnicity-specific and shared pathogenic variants between different ethnicities. This figure displays the minor allele frequency (MAF) of the five most frequent pathogenic variants identified in the gnomAD population, across different ethnicities that were classified as pathogenic/likely pathogenic.

The five most frequent pathogenic variants associated with VWD among the 321 variants for each ethnicity identified in gnomAD are shown in Table 3. Several variants, including p.Ala631Val, p.Arg854Gln, p.Tyr1584Cys, p.Pro1266Leu, and p.Pro1266Gln were shared across multiple ethnicities (Fig. 3).

**Table 3:** Five most frequent pathogenic variants stratified by ethnicity.

	cDNA	Protein	rs ID	Type of variant	MAF	VWD type of variant
African/African American	c.1892 C > T	p.Ala631Val	rs199963222	missense	0.001907812	Type 1
	c.2561G > A	p.Arg854Gln	rs41276738	missense	0.000985747	Type 2N
	c.4024 C > T	p.Arg1342Cys	rs61749404	missense	0.000826094	Type 1
	c.4751 A > G	p.Tyr1584Cys	rs1800386	missense	0.000466816	Type 1
	c.2344 C > T	p.Arg782Trp	rs61748471	missense	0.00045383	Type 2N
Admixed American	c.2561G > A	p.Arg854Gln	rs41276738	missense	0.002532152	Type 2N
	c.4751 A > G	p.Tyr1584Cys	rs1800386	missense	0.00150005	Type 1
	c.1892 C > T	p.Ala631Val	rs199963222	missense	0.001289887	Type 1
	c.3797 C > T	p.Pro1266Leu	rs61749370	missense	0.001033299	Type 2B
	c.250 C > T	p.Leu84Phe	rs372664002	missense	0.000783516	Type 2A
East Asian	c.3797 C > T	p.Pro1266Leu	rs61749370	missense	0.00331193	Type 2B
	c.4751 A > G	p.Tyr1584Cys	rs1800386	missense	0.003006554	Type 1
	c.2561G > A	p.Arg854Gln	rs41276738	missense	0.001317212	Type 2N
	c.3613 C > T	p.Arg1205Cys	rs373787920	missense	0.000337747	Type 1
	c.7135 C > T	p.Arg2379Cys	rs61751283	missense	0.000135117	Type 1
Ashkenazi Jewish	c.5014G > A	p.Gly1672Arg	rs61750598	missense	0.001852017	Type 2A
	c.6311 C > T	p.Thr2104Ile	rs61750616	missense	0.001403431	Type 1
	c.2303G > A	p.Arg768Gln	rs772203447	missense	0.000267368	Type 1
	c.3923G > A	p.Arg1308His	rs61749388	missense	0.000245185	Type 2A
	c.3797 C > A	p.Pro1266Gln	rs61749370	missense	0.00022648	Type 2M
European Finnish	c.2561G > A	p.Arg854Gln	rs41276738	missense	0.005450059	Type 2N
	c.3797 C > T	p.Pro1266Leu	rs61749370	missense	0.004452568	Type 2B
	c.4751 A > G	p.Tyr1584Cys	rs1800386	missense	0.004016504	Type 1
	c.5014G > A	p.Gly1672Arg	rs61750598	missense	0.000801635	Type 2A
	c.4975 C > T	p.Arg1659Ter	rs61750595	Stop-gained	0.000548194	Type 3
Middle Eastern	c.4751 A > G	p.Tyr1584Cys	rs1800386	missense	0.001986097	Type 1
	c.1892 C > T	p.Ala631Val	rs199963222	missense	0.001657688	Type 1
	c.3797 C > A	p.Pro1266Gln	rs61749370	missense	0.00148662	Type 2M
	c.2561G > A	p.Arg854Gln	rs41276738	missense	0.00082481	Type 2N
	c.4255 C > A	p.His1419Asn	rs375498783	missense	0.000822639	Type 2A
European (non-Finnish)	c.2561G > A	p.Arg854Gln	rs41276738	missense	0.005974445	Type 2N
	c.4751 A > G	p.Tyr1584Cys	rs1800386	missense	0.004104996	Type 1
	c.3797 C > T	p.Pro1266Leu	rs61749370	missense	0.000447809	Type 2B
	c.3797 C > A	p.Pro1266Gln	rs61749370	missense	0.000352645	Type 2M
	c.706 C > T	p.Arg236Cys	rs140912382	missense	0.000281346	Type 1
South Asian	c.3797 C > A	p.Pro1266Gln	rs61749370	missense	0.00385638	Type 2M
	c.5793G > C	p.Gln1931His	rs574811308	missense	0.002319854	Type 3
	c.1309G > T	p.Asp437Tyr	rs375486035	missense	0.000768504	Type 3
	c.5347T > G	p.Ser1783Ala	rs267607353	missense	0.00069176	Type 2M
	c.2561G > A	p.Arg854Gln	rs41276738	missense	0.000636789	Type 2N
Remaining	c.2561G > A	p.Arg854Gln	rs41276738	missense	0.004304636	Type 2N
	c.4751 A > G	p.Tyr1584Cys	rs1800386	missense	0.002902391	Type 1
	c.3797 C > T	p.Pro1266Leu	rs61749370	missense	0.000804873	Type 2B
	c.3797 C > A	p.Pro1266Gln	rs61749370	missense	0.000773432	Type 2M
	c.1892 C > T	p.Ala631Val	rs199963222	missense	0.000694904	Type 1

p.Gly1672Arg appeared among the top five frequent variants in Ashkenazi Jewish and European Finnish populations. However, several variants were ethnicity-specific: p.Arg1342Cys and p.Arg782Trp in African/African American; p.Leu84Phe in Admixed American; p.Arg1205Cys and p.Arg2379Cys in East Asian; p.Thr2104Ile, p.Arg768Gln, and p.Arg1308His in Ashkenazi Jewish; p.Arg1659Ter in European Finnish; p.His1419Asn in Middle Eastern; p.Arg236Cys in European; and p.Gln1931His, p.Asp437Tyr, and p.Ser1783Ala in South Asian. Notably, the p.Ser1731Thr and p.Arg1399His variants—both associated with type 2M VWD characterized by collagen-binding defects^13,14^ —were the most frequent in the Ashkenazi Jewish population (for both variants) and in the European and Finnish populations (for p.Arg1399His). However, due to their MAFs (~ 2% and ~ 1.3%, respectively), they were excluded from our prevalence analysis.

### Domain distribution of VWF variants

**Fig. 4:**
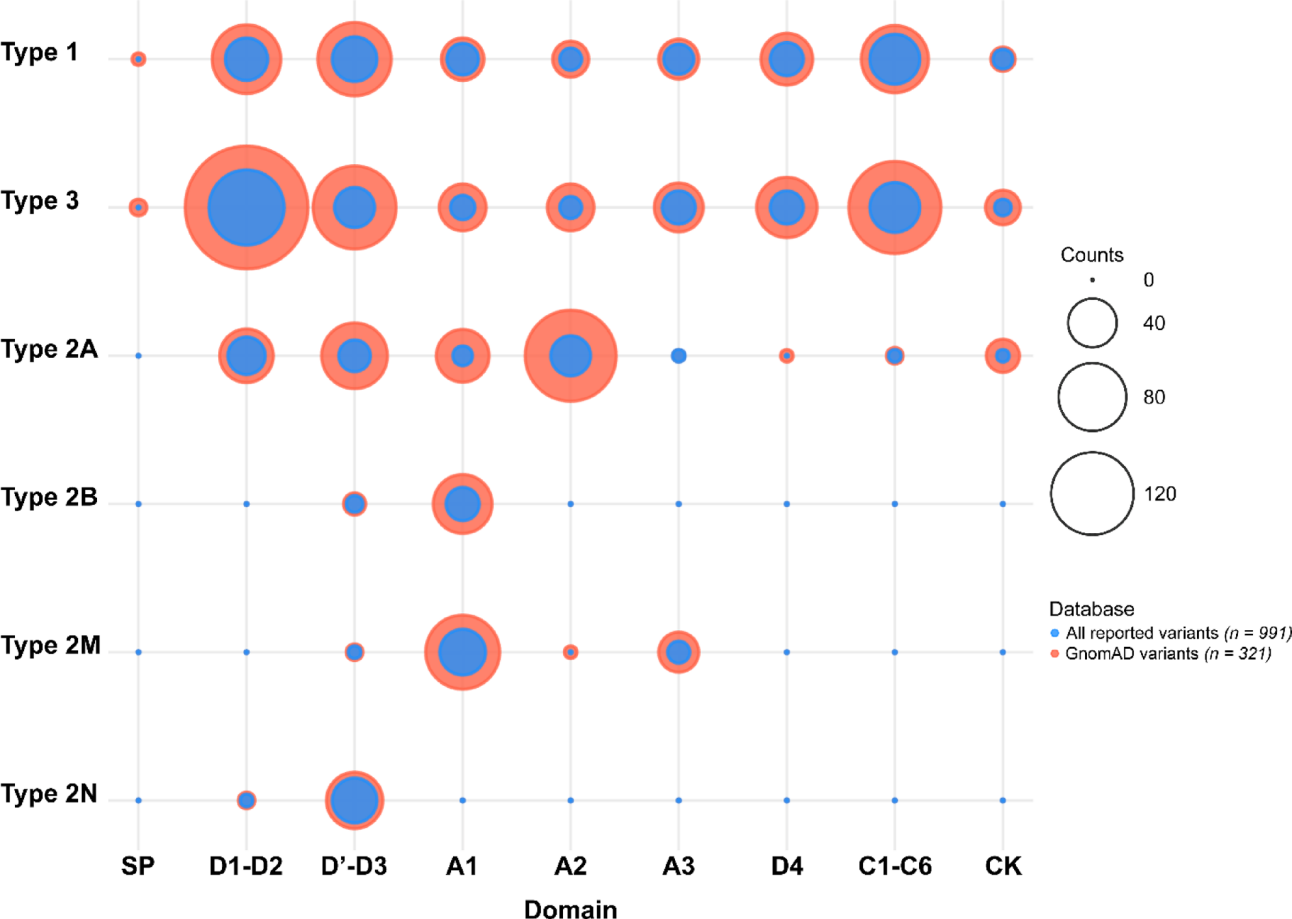
The domain distribution of all reported *VWF* pathogenic variants and the pathogenic variants selected from gnomAD. We identified 991 pathogenic *VWF* variants (including single nucleotide variants and short insertions/deletions) that have been reported to cause von Willebrand disease (VWD), of which 321 are present in the gnomAD population. We found that while variants associated with types 1 and 3 are spread across the entire *VWF* gene, type 2 variants are concentrated within specific functional domains of the gene.

The domain distributions of all *VWF* variants reported in the HGMD, LOVD and ClinVar datasets and literature (*n* = 991) and the pathogenic variants selected from gnomAD (*n* = 321) are shown in Fig. 4. As previously reported^1^, we found that while variants associated with types 1 and 3 are distributed across the entire *VWF*, type 2 variants are localized within specific functional domains of VWF. This pattern holds true for both the 991 previously reported variants in VWD, and the 321 (previously reported) pathogenic variants identified in gnomAD (Fig. 4).

### Distribution of VWD types in VWF gene variants

**Fig. 5:**
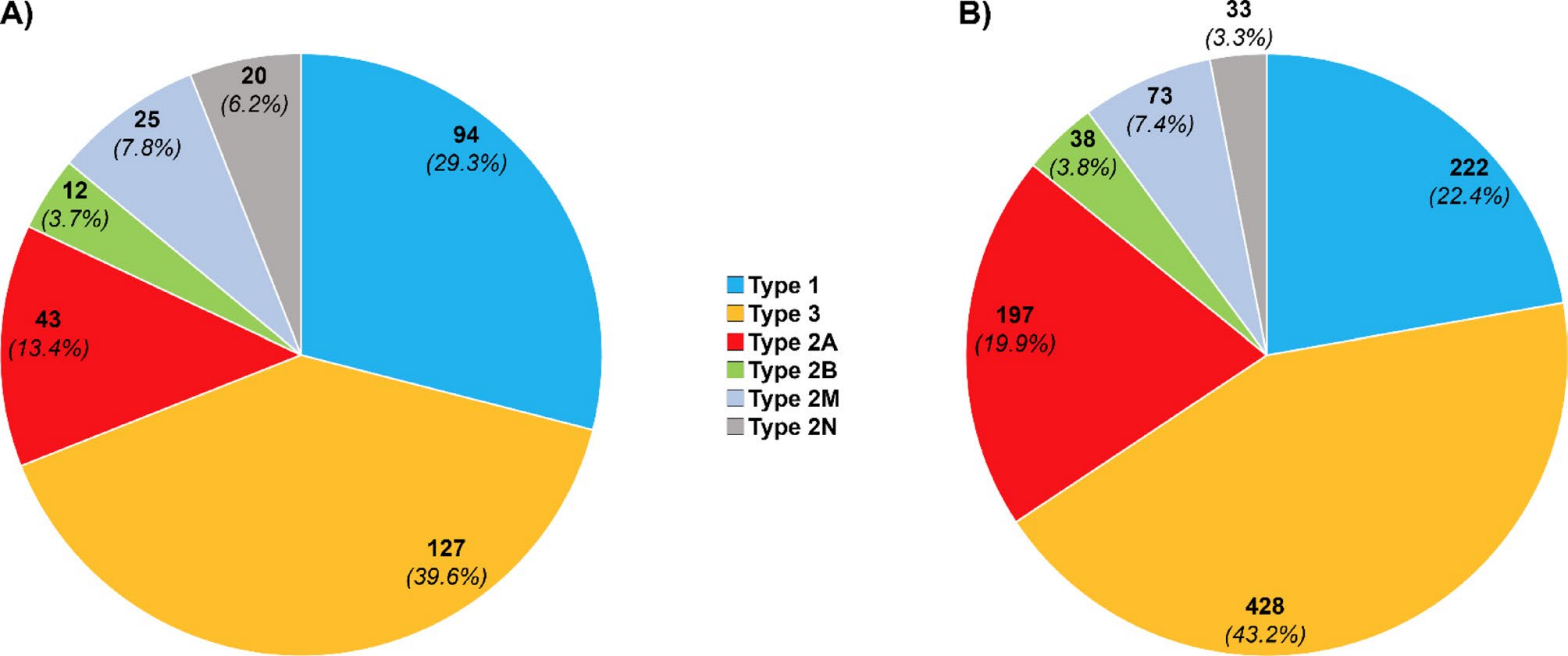
Distribution of von Willebrand disease (VWD) types in VWF gene variants. (A) Among gnomAD-derived pathogenic variants, most were associated with type 3 VWD, followed by types 1, 2A, 2M, 2N, and 2B. (B) A similar pattern was observed among 991 previously reported variants, with the highest number linked to type 3 VWD, followed by types 1, 2A, 2M, 2B, and 2N.

Among the VWD-causing variants identified in gnomAD (*n* = 321), 127 were associated with type 3, 94 with type 1, 43 with type 2A, 25 with type 2M, 20 with type 2N, and 12 with type 2B (Fig. 5A). A similar pattern was observed among the 991 variants reported in association with VWD: 428 were reported in type 3 patients, 222 in type 1, 197 in 2A, 73 in 2M, 38 in 2B, and 33 in 2N (Fig. 5B).

## Discussion

The prevalence of genetic diseases has traditionally been determined through direct observation of the disease or using disease registries. Three seminal studies have estimated the prevalence of VWD in countries such as Italy^5^, the U.S ^6^, and Canada^7^, with prevalences ranging from 0.6% to 1.3%, and approximately 1 in 1,000 individuals showed clinical manifestations. However, it is important to emphasize that these studies were constrained by relatively small sample sizes and geographic limitations, and because none of these included genetic analyses, the burden of the disorder might be underestimated. Recently, using the data from the WFH Annual Global Survey (2022) and national registries an estimated global VWD prevalence of 25.6 (± 48.8) per million people was reported^15^. Due to the registry-based nature of this report and the limitations associated with estimating prevalence from such data, it is likely that VWD is substantially underestimated. Previous literature^16–18^ has pointed out that registry data may not capture undiagnosed or misdiagnosed cases, especially in regions with limited healthcare access or insufficient diagnostic facilities. Moreover, registry data often rely on reported cases, which may exclude individuals with milder forms of VWD or those who have not sought medical attention. As a result, the global prevalence of VWD is likely to be much higher than suggested by current estimates. By utilizing genetic epidemiology methods that provide valid prevalence estimations on a population scale, we are able to establish more accurately the prevalence of genetic disorders. The advent of NGS has revolutionized genetic epidemiology, as data from large international consortia are increasingly accessible^9,11^. This enables the estimation of prevalence based on the allele frequency of pathogenic variants. We have previously estimated the global and ethnicity-specific prevalence of such disorders as VWD (gnomAD v2)^10^, platelet-type VWD (gnomAD v4.1)^19^, hereditary thrombotic thrombocytopenic purpura (gnomAD v4.1)^20^ and our findings suggested a significantly higher prevalence than earlier reports.

The recently released gnomAD v4.1 includes data from 807,162 individuals—nearly five times the sample size of previous combined releases (v2 and v3). Our genetic epidemiology investigation provides a global and within-population estimation of VWD prevalence using genome and exome sequencing data from these 807,162 individuals. Based on the allele frequency of 321 documented pathogenic variants in *VWF*, the worldwide prevalence of VWD was estimated as nearly 11 per 1,000 for type 1, 1.3 per 1,000 for type 2A, 1.7 per 1,000 for type 2B, and 1.5 per 1,000 for type 2M. The global prevalence for the recessive forms of VWD was estimated to be nearly 34 cases per million for type 2N and 1.3 cases per million for type 3. Importantly, including loss-of-function SVs and CNVs from gnomAD slightly raised the estimated prevalence of type 3 VWD to 1.8 per million. This suggests that in a global population of about 8 billion people, the estimated prevalences of different VWD types are as follows: 88 million individuals with type 1, 10.4 million with type 2A, 13.6 million with type 2B, 12 million with type 2M, 272,000 with type 2N, and 14,400 with type 3.

These data suggest that VWD is likely grossly underdiagnosed worldwide. Although VWD is common, available data indicates that it is often underdiagnosed^8,21,22^. This paradox can be attributed to several factors, including the complexity of diagnosis, unavailability of all the diagnostic tools, challenges in differentiating between normal and abnormal bleeding symptoms, overlapping symptoms with other conditions, the mild clinical severity of some VWD types, and limited awareness of the disease among non-specialist healthcare providers and patients. This delayed diagnosis of VWD can indeed lead to chronic health issues, psychological impact, increased burden on healthcare systems, and increased risk of complications, all of which emphasize the importance of early recognition and in turn proper management of affected patients^1,23^.

The prevalence of different VWD types (worldwide and among specific ethnicities) has not yet been established. An additional important finding from our study is that the prevalence of different VWD types varies across populations (Table 2). Type 1 was the most common phenotype, affecting up to 1.1% of the general population, with similar frequencies across most ethnicities, though it was most prevalent in the European population. We found a generally consistent global prevalence among types 2A, 2B, and 2M; however, different populations exhibited varying frequencies for each of these phenotypes. Type 2N was the second rarest phenotype of VWD, being extremely rare in East Asian populations (0.1 per 10^6^) but more common in non-Finnish and Finnish European populations (46,2 and 44,7 per 10^6^), primarily due to the p.Arg854Gln variant. Type 3 was the rarest phenotype, with prevalence ranging from 0.01 per 10^6^ in Ashkenazi Jewish populations to 19.5 per 10^6^ in South Asian populations. This finding underscores the importance of ethnic background in VWD prevalence studies.

An example that supports the reliability of our data is the prevalence of type 2N. Previous studies have shown that homozygosity for p.Arg854Gln causes type 2N^24,25^, and we identified 26 homozygous cases in the 807,162 gnomAD population. This corresponds to 32 cases of type 2N per million people. Using the Hardy-Weinberg equation, our estimation of 34 cases per million closely aligns with this finding, further validating our method and results.

The prevalence of VWD may be even higher than our estimate. One reason for this is that we excluded variants with a MAF > 1%, despite their association with VWD, in order to avoid overestimating the prevalence. For example, the p.Ser1731Thr and p.Arg1399His variants are reported to be associated with type 2M characterized by collagen binding defects, supported by experimental studies and reported in type 2M patients from multiple expert labs^13,14,26–28^. We found that they have MAFs of 2% and 1.5% in Ashkenazi Jewish individuals and p.Arg1399His has a frequency up to 1.3% in the European and Finnish populations, thus were excluded from our analysis. Including the p.Ser1731Thr variant alone significantly increases the estimated prevalence of type 2M from 1.5 to 3.8 cases per 1000, with a frequency up to 42 per 1000 in Ashkenazi Jewish ethnicity. Additionally, we did not include previously reported variants that play a small role in reducing VWF levels, or those with incomplete penetrance, which are observed in cases with borderline VWF plasma levels. Although these variants may cause a mild VWD phenotype or influence the manifestations of VWD in moderately to severe affected patients, particularly in those with low VWF or type 1 VWD and VWF levels between 30 and 50 IU/dL. Structural variants, including CNVs, contribute to VWD, particularly in type 3 and some type 1 cases^29,30^. These often involve large deletions or duplications in the *VWF* that may be missed by standard sequencing methods. Since such variants were not considered in our analysis for type 1 VWD, the true prevalence is likely higher than our estimate.

A key strength of this study is the use of the comprehensive gnomAD v4 dataset, which offers unprecedented population-scale resolution. By applying rigorous variant classification through ACMG/AMP guidelines, ClinVar annotations, and in silico predictive tools, the analysis provides a robust and systematic estimate of VWD prevalence grounded in clinically relevant and biologically informed criteria.

There are some limitations in the present study. Some *VWF* variants associated with VWD may not exhibit full penetrance, potentially affecting the observed prevalence estimates. However, specifically for type 1, we used only variants that were found in type 1 patients with VWF levels < 30 IU/dL. Biological samples of gnomAD population were not available. The analysis may have underestimated the number of potentially pathogenic variants, including those in promoter regions, deep intronic sequences, large insertions/deletions, and gene rearrangements. This is more likely in cases of VWD phenotypes caused by CNVs or SVs, including type 3 and certain forms of type 1. Furthermore, gnomAD v4.1 incorporates data from UK Biobank and other large cohorts, each with inherent recruitment biases. These include the healthy volunteer bias, depletion of severe pediatric disorders, cryptic relatedness, ancestry misclassification, and the underrepresentation of some populations (e.g., Middle Eastern, *n* = 3,031). Collectively, these factors suggest that the “true” VWD prevalence is likely even higher than estimated in this study.

In conclusion, we have estimated the worldwide and within-population prevalence of VWD using available genome and exome sequencing data from over 800,000 individuals in the gnomAD database. Our study reveals a significantly higher global prevalence of VWD than previously reported. We also determined the global and within-population prevalence of different VWD types across various ethnicities. These findings suggest that a substantial number of VWD patients remain undiagnosed, potentially leading to undertreatment.

## Methods

### *VWF* gene variants in the population-based gnomAD

We extracted and analyzed all variants in the *VWF* (ENST00000261405.10, NM_000552.5) from the gnomAD population (v4.1), which includes 730,947 whole exomes (416,555 individuals from the UK Biobank) and 76,215 genomes. Date then were annotated in ANNOVAR (https://annovar.openbioinformatics.org/). This gnomAD dataset, derived from genetic studies of diverse populations, represents a total of 807,162 individuals. The gnomAD sequencing data are aligned to the GRCh38 human reference genome and include 8 major ethnicities: African/African American, Admixed American, Ashkenazi Jewish, East Asian, Finnish, Middle Eastern, non-Finnish European, and South Asian. This broad representation makes it ideal for estimating the prevalence of genetic diseases at both global and ethnic group. The dataset underwent extensive quality control by gnomAD investigators to exclude low-quality sequences and flag variants with questionable reliability. Only *VWF* variants that passed these quality controls (using the gnomAD random forest filters) were included in the present analysis. Due to limitations of NGS in detecting large insertions, duplications, deletions, and complex rearrangements, our analysis primarily focused on single nucleotide variants (SNVs) and short insertions/deletions reported to cause all VWD types. Nevertheless, for type 3 VWD, we performed an additional analysis that also included SVs and CNVs found in the gnomAD population and predicted to result in loss of function.

### Filtering processes

Among the *VWF* variants identified in the 807,162 gnomAD population, we considered the following: all variants reported to be associated with VWD in the Human Gene Mutation Database (HGMD, https://www.hgmd.cf.ac.uk/ac/index.ph) and/or the Leiden Open Variation Database (LOVD, https://databases.lovd.nl/shared/genes/VWF/) and/or ClinVar (https://www.ncbi.nlm.nih.gov/clinvar/?term=%22VWF%22%5BGENE%5D&redir=gene) and/or PubMed (https://pubmed.ncbi.nlm.nih.gov/). All these previously reported variants associated with VWD were assessed using ClinVar classifications; variants classified as Pathogenic or Likely pathogenic were selected. Variants with uncertain significance, conflicting classifications, or lacking ClinVar annotation were evaluated using ACMG criteria via GeneBe (https://genebe.net/) and Franklin (https://franklin.genoox.com/clinical-db/home). Again, variants classified as Pathogenic or Likely pathogenic by these tools were selected. For variants still classified as of uncertain significance, in silico tools including CADD score (Combined Annotation Dependent Depletion; https://cadd.gs.washington.edu/score) and SpliceAI (https://spliceailookup.broadinstitute.org/) were used. If these tools predicted pathogenicity, the variants were retained; if not, the original articles reporting the variants were reviewed to verify their disease-causing nature. Regarding SVs, we selected those gnomAD variants that resulted in loss of function, while gnomAD CNVs were evaluated using AnnotSV (https://lbgi.fr/AnnotSV/) and were all classified as pathogenic.

### Calculation of VWD prevalence

The global and population-specific prevalence of VWD was calculated based on the allele frequencies of all *VWF* variants identified in gnomAD that had been previously reported to cause VWD in genetic databases (HGMD, LOVD, or ClinVar) or in the literature, and that passed our filtering criteria. We calculated the estimated prevalence using the Hardy-Weinberg equation (p^2^ + 2pq + q^2^ = 1), where p is the population frequency of the major allele, 2pq is the incidence of an autosomal dominant condition, and q is the population frequency of the minor allele. For multi-allelic recessive VWD types (2N and 3), prevalence was estimated using the following formula for multiple pathogenic alleles:

$$\:{Q}^{2}=\:{\left.\left({\sum\:}_{i}{q}_{i}\right.\right)}^{2}=\:{\sum\:}_{i}{q}_{i}^{2}+\:2{\sum\:}_{\left\{i<j\right\}{q}_{i}}{q}_{j}$$

This method explicitly accounts for both homozygous and compound heterozygous genotypes across all pathogenic alleles. For all prevalence estimates, 95% confidence intervals (CIs) were calculated using Poisson-based methods, which are appropriate for rare events and low-frequency variants.

It is important to emphasize that, due to the variable penetrance and expression of the type 1 VWD phenotype, we only included previously reported type 1 variants associated with VWF levels < 30 IU/dL in our prevalence calculation for type 1. For type 2N, we used the allele frequency of all missense variants previously linked to this type, along with null variants associated with type 1 and type 3. In the case of type 3, we considered the allele frequency of all variants reported to cause type 3, as well as null variants from type 1. An additional analysis for type 3 VWD was performed to include SVs and CNVs identified in the gnomAD population that were predicted to result in loss of function. For types 2A, 2B, and 2M, we included the allele frequency of previously reported variants causing these phenotypes. Furthermore, variants with a minor allele frequency (MAF) ≥ 1%, despite their confirmed association with VWD, were excluded from our prevalence calculation.

## Supplementary Information

Below is the link to the electronic supplementary material.

Supplementary Material 1

## Data Availability

Public datasets used in this study are described in the Methods section. All relevant data are provided within the manuscript. Additional information can be requested from the corresponding authors at omid.seidizadeh@unimi.it and [flora.peyvandi@unimi.it](mailto: flora.peyvandi@unimi.it) .
