# Supplementary material for: Updated global prevalence and ethnic diversity of von Willebrand disease based on population genetics analysis
Source: Sci Rep. 2026 Jan 20;16:5824. doi: 10.1038/s41598-026-36145-6 (PMC12894688; doi:10.1038/s41598-026-36145-6)
Supplement: Supplementary file 1 — Supplementary Material 1 [file 41598_2026_36145_MOESM1_ESM.pdf]

Updated global prevalence and ethnic diversity of von Willebrand disease based on population genetics analysis

Omid Seidizadeh[1,2], Andrea Cairo[2], Camilla Oriani[2], Flora Peyvandi[1, 2]

1. Università degli Studi di Milano. Department of Pathophysiology and Transplantation, Milan, Italy
2. Fondazione IRCCS Ca'Granda Ospedale Maggiore Policlinico. Angelo Bianchi Bonomi Hemophilia and Thrombosis Center, Milan, Italy

Correspondence

Omid Seidizadeh, Ph.D., and Flora Peyvandi, M.D., Ph.D.,

Fondazione IRCCS Ca'Granda Ospedale Maggiore Policlinico, Angelo Bianchi Bonomi Hemophilia and Thrombosis Center, Department of Pathophysiology and Transplantation, Università degli Studi di Milano, Via Pace 9, 20122 Milan, Italy.

Emails: omid.seidizadeh@unimi.it, flora.peyvandi@unimi.it

Supplementary Materials

Table: 8
Figure: 1

Supplementary Table S1. Identified pathogenic variants in the gnomAD that were reported to be associated with von Willebrand disease (VWD).

Transcript Consequence	Protein Consequence	rsIDs	Type of variant	Allele Frequency	Clinical classification	CLINVAR	ACGM
c.8411G>A	p.Cys2804Tyr	rs267607370	missense	1.85895E-06	type3	not provided	Likely pathogenic
c.8357T>C	p.Leu2786Pro	NA	missense	1.23897E-06	type1	not provided	Likely pathogenic
c.8347C>T	p.Gln2783Ter	rs1298396777	stop gained	1.85845E-06	type1	not provided	Uncertain significance
c.8327C>T	p.Pro2776Leu	rs61751312	missense	1.54884E-05	type1	not provided	Uncertain significance
c.8324C>G	p.Ser2775Cys	NA	missense	6.19482E-07	type2A	not provided	Uncertain significance
c.8216G>A	p.Cys2739Tyr	rs61751305	missense	1.23902E-06	type3	Likely pathogenic	Likely pathogenic
c.8215T>C	p.Cys2739Arg	rs1591827147	missense	6.19554E-07	type1	Uncertain significance	Likely pathogenic
c.8159del	p.Glu2720GlyfsTer24	NA	frameshift	6.19823E-07	type3	not provided	Uncertain significance
c.8155+6T>C	NA	rs1223422347	intron	1.3629E-05	type1	Conflicting classifications of pathogenicity	Uncertain significance
c.8148T>A	p.Cys2716Ter	rs1565806605	stop gained	6.19487E-07	type1	not provided	Pathogenic
c.8078G>A	p.Cys2693Tyr	rs62641243	missense	6.19543E-07	type1	not provided	Likely pathogenic
c.8012G>A	p.Cys2671Tyr	rs61751303	missense	6.19587E-07	type3	Likely pathogenic	Likely pathogenic
c.7987C>T	p.Arg2663Cys	rs370662678	missense	5.26848E-05	type1	Pathogenic/Likely pathogenic	Likely pathogenic
c.7985A>G	p.Lys2662Arg	rs1411072226	missense	6.19636E-07	type1	not provided	Uncertain significance
c.7887+2T>A	NA	rs113814258	splice	3.84169E-05	type3	Conflicting classifications of pathogenicity	Uncertain significance
c.7886del	p.Leu2629ArgfsTer25	NA	frameshift	1.23908E-06	type3	Pathogenic	Pathogenic
c.7856G>A	p.Cys2619Tyr	NA	missense	6.19509E-07	type1	not provided	Likely pathogenic
c.7770+1G>A	NA	rs200770256	splice	6.19524E-07	type1	not provided	Pathogenic
c.7730-1G>C	NA	rs267607366	splice	6.1955E-07	type3	Likely pathogenic	Pathogenic
c.7729+7C>T	NA	rs61751301	splice	6.20787E-07	type3	Likely pathogenic	Uncertain significance
c.7729+5G>A	NA	rs918057829	intron	6.20652E-06	type1	Uncertain significance	Uncertain significance
c.7670G>A	p.Cys2557Tyr	rs774929265	missense	6.19496E-07	type1	not provided	Uncertain significance
c.7651C>T	p.Gln2551Ter	rs776340794	stop gained	6.19519E-07	type3	not provided	Pathogenic
c.7636A>T	p.Asn2546Tyr	rs61751298	missense	3.71742E-06	type3	Conflicting classifications of pathogenicity	Uncertain significance
c.7630C>T	p.Gln2544Ter	rs61751297	stop gained	6.19497E-07	type3	Pathogenic	Pathogenic
c.7627C>T	p.Gln2543Ter	rs1368264172	stop gained	8.0541E-06	type1	not provided	Likely pathogenic
c.7604G>A	p.Arg2535Gln	rs137987906	missense	9.16873E-05	type3	Uncertain significance	Uncertain significance
c.7603C>T	p.Arg2535Ter	rs61751296	stop gained	2.60192E-05	type3	Pathogenic	Pathogenic
c.7558C>T	p.Gln2520Ter	rs1448479214	stop gained	1.23909E-06	type3	not provided	Pathogenic
c.7552G>A	p.Gly2518Ser	rs61751293	missense	1.05323E-05	type1	Uncertain significance	Uncertain significance
c.7524 7525del	p.Asp2509LeufsTer23	rs1591836930	frameshift	3.09917E-06	type3	Pathogenic	Pathogenic

c.7524 7525del	p.Asp2509LeufsTer23	rs1591836930	frameshift	3.09917E-06	type3	Pathogenic	Pathogenic
c.7489T>C	p.Ser2497Pro	rs61751292	missense	1.11517E-05	type1	not provided	Uncertain significance
c.7471T>C	p.Cys2491Arg	NA	missense	6.1949E-07	type3	not provided	Likely pathogenic
c.7464C>T	p.Gly2488Gly	rs900907976	synonymous	1.11514E-05	type3	Likely pathogenic	Uncertain significance
c.7437G>A	p.Ser2479Ser	rs267607363	splice	7.43661E-06	type3	Pathogenic	Uncertain significance
c.7436C>A	p.Ser2479Ter	rs771209761	stop gained	6.19723E-07	type1	not provided	Pathogenic
c.7408C>T	p.Gln2470Ter	rs61751288	stop gained	3.34574E-05	type1	Pathogenic/Likely pathogenic	Pathogenic
c.7393G>A	p.Val2465Met	rs375655409	missense	6.19545E-05	type2A	Likely pathogenic	Uncertain significance
c.7390C>T	p.Arg2464Cys	rs61751286	missense	0.000200733	type1	Pathogenic/Likely pathogenic	Pathogenic
c.7361C>A	p.Thr2454Asn	rs200486416	missense	4.33666E-06	type3	Uncertain significance	Uncertain significance
c.7344C>A	p.Cys2448Ter	NA	stop gained	1.23908E-06	type3	not provided	Pathogenic
c.7333del	p.Glu2445ArgfsTer16	NA	frameshift	6.1956E-07	type3	not provided	Pathogenic
c.7300C>T	p.Arg2434Ter	rs62643640	stop gained	3.71757E-06	type3	Pathogenic	Likely pathogenic
c.7288-2A>G	NA	NA	splice	1.85866E-06	type1	not provided	Uncertain significance
c.7287+1G>C	NA	rs1235191685	splice	6.19558E-07	type3	Uncertain significance	Pathogenic
c.7135C>T	p.Arg2379Cys	rs61751283	missense	7.43735E-05	type1	Uncertain significance	Uncertain significance
c.7085G>T	p.Cys2362Phe	rs61750630	missense	2.47904E-06	type3	Pathogenic	Likely pathogenic
c.7080C>A	p.Cys2360Ter	rs111597150	stop gained	6.1981E-07	type1	Pathogenic	Pathogenic
c.7056C>T	p.Gly2352Gly	rs746482504	synonymous	4.9572E-06	type1	Likely pathogenic	Uncertain significance
c.7028G>T	p.Gly2343Val	rs61750629	missense	9.91282E-06	type1	not provided	Uncertain significance
c.6973T>A	p.Cys2325Ser	rs1256082707	missense	6.19541E-07	type3	Likely pathogenic	Pathogenic
c.6911G>A	p.Cys2304Tyr	rs61750626	missense	1.23901E-06	type1	Pathogenic/Likely pathogenic	Pathogenic
c.6853 6856del	p.Ser2285GlyfsTer24	NA	frameshift	6.19519E-07	type1	not provided	Pathogenic
c.6847T>C	p.Cys2283Arg	rs2136376158	missense	6.19512E-07	type3	Likely pathogenic	Pathogenic
c.6798+1G>T	NA	rs61750624	splice	6.19612E-06	type1	Likely pathogenic	Likely pathogenic
c.6743G>A	p.Cys2248Tyr	rs2136382972	missense	1.23896E-06	type3	Pathogenic	Likely pathogenic
c.6709T>C	p.Cys2237Arg	rs770625592	missense	3.71693E-06	type3	not provided	Uncertain significance
c.6697G>A	p.Glu2233Lys	rs61750623	missense	1.8585E-06	type3	Uncertain significance	Uncertain significance
c.6634T>C	p.Cys2212Arg	rs1943750562	missense	1.85861E-06	type3	Uncertain significance	Likely pathogenic
c.6553C>T	p.Arg2185Trp	rs569962285	missense	4.27533E-05	type1	Uncertain significance	Uncertain significance
c.6551G>A	p.Cys2184Tyr	rs2136385288	missense	1.23917E-06	type1	Likely pathogenic	Pathogenic
c.6536C>T	p.Ser2179Phe	rs61750620	missense	6.19551E-07	type1	Likely pathogenic	Likely pathogenic
c.6479A>G	p.Tyr2160Cys	rs779764302	missense	6.81482E-06	type1	Uncertain significance	Uncertain significance
c.6385G>T	p.Glu2129Ter	rs61750617	stop gained	6.19504E-07	type3	Pathogenic	Pathogenic
c.6311C>T	p.Thr2104Ile	rs61750616	missense	3.96533E-05	type1	Uncertain significance	Uncertain significance

c.6277G>C	p.Ala2093Pro	rs149274687	missense	1.17723E-05	type1	not provided	Uncertain significance
c.6182del	p.Phe2061SerfsTer38	rs61750614	frameshift	1.23903E-06	type3	Pathogenic	Pathogenic
c.5941G>T	p.Glu1981Ter	rs61750613	stop gained	1.23905E-06	type3	not provided	Pathogenic
c.5849G>A	p.Cys1950Tyr	rs1591849732	missense	3.1023E-06	type1	Uncertain significance	Uncertain significance
c.5842+1G>C	NA	NA	splice	6.22581E-07	type3	not provided	Pathogenic
c.5801T>G	p.Val1934Gly	rs139845585	missense	6.26024E-05	type1	Conflicting classifications of pathogenicity	Uncertain significance
c.5793G>C	p.Gln1931His	rs574811308	missense	0.00013634	type3	Pathogenic	Uncertain significance
c.5791C>T	p.Gln1931Ter	rs1359172781	stop gained	6.19774E-07	type3	Pathogenic	Pathogenic
c.5695T>C	p.Cys1899Arg	rs559785610	missense	6.19884E-07	type1	not provided	Uncertain significance
c.5636G>T	p.Cys1879Phe	NA	missense	6.19565E-07	type1	not provided	Uncertain significance
c.5621-2A>C	NA	rs769632868	splice	4.95654E-06	type3	not provided	Likely pathogenic
c.5614 5615del	p.Cys1872LeufsTer5	rs1944020823	frameshift	6.19553E-07	type3	not provided	Pathogenic
c.5557C>T	p.Arg1853Ter	rs61750612	stop gained	1.42506E-05	type3	Pathogenic	Pathogenic
c.5509C>T	p.Arg1837Trp	rs1250634491	missense	2.72648E-05	type1	Uncertain significance	Uncertain significance
c.5483G>T	p.Gly1828Val	rs1299445904	missense	6.1983E-07	type3	Uncertain significance	Likely pathogenic
c.5455+1G>A	NA	rs2136408263	splice	1.23907E-06	type3	Pathogenic	Likely pathogenic
c.5380A>G	p.Lys1794Glu	rs267607355	missense	1.23905E-06	type1	Pathogenic	Likely pathogenic
c.5368C>T	p.Pro1790Ser	rs1944054577	missense	6.19566E-07	type2A	not provided	Uncertain significance
c.5347T>G	p.Ser1783Ala	rs267607353	missense	4.39854E-05	type2M	Uncertain significance	Uncertain significance
c.5342T>G	p.Leu1781Trp	NA	missense	6.19499E-07	type3	not provided	Uncertain significance
c.5335C>T	p.Arg1779Ter	rs61750606	stop gained	9.29363E-06	type1	Pathogenic	Pathogenic
c.5321T>C	p.Leu1774Ser	rs61750605	missense	1.23901E-06	type1	Likely pathogenic	Likely pathogenic
c.5312-2 5312-1del	NA	rs1287088175	splice	1.23909E-06	type1	Conflicting classifications of pathogenicity	Likely pathogenic
c.5311G>A	p.Gly1771Arg	rs370016586	missense	1.30137E-05	type3	Uncertain significance	Uncertain significance
c.5282T>A	p.Met1761Lys	rs1490774216	missense	2.29236E-05	type2M	not provided	Uncertain significance
c.5273T>C	p.Val1758Ala	rs1375213244	missense	6.19498E-07	type1	not provided	Uncertain significance
c.5235G>T	p.Trp1745Cys	rs267607352	missense	6.19509E-07	type2M	Likely pathogenic	Likely pathogenic
c.5200C>T	p.Gln1734Ter	rs374707563	stop gained	1.23901E-05	type3	not provided	Likely pathogenic
c.5192C>T	p.Ser1731Leu	rs764077750	missense	6.19543E-06	type2M	Uncertain significance	Uncertain significance
c.5180 5181insTT	p.Thr1728SerfsTer29	rs61750602	frameshift	1.85863E-06	type1	not provided	Pathogenic
c.5170+1G>A	NA	rs764543553	splice	1.23897E-06	type3	Likely pathogenic	Likely pathogenic
c.5085 5087del	p.Leu1696del	rs2136409523	inframe deletion	6.19546E-07	type3	Uncertain significance	Uncertain significance
c.5053+3A>G	NA	rs1281433274	intron	1.86517E-06	type1	not provided	Uncertain significance
c.5014G>A	p.Gly1672Arg	rs61750598	missense	0.000187935	type2A	Uncertain significance	Uncertain significance

c.4975C>T	p.Arg1659Ter	rs61750595	stop gained	3.22381E-05	type3	Pathogenic	Pathogenic
c.4969C>A	p.Leu1657Ile	rs61750592	missense	6.19882E-07	type2A	not provided	Likely pathogenic
c.4946T>A	p.Ile1649Asn	NA	missense	6.19706E-07	type2A	not provided	Likely pathogenic
c.4931G>A	p.Trp1644Ter	rs1591862022	stop gained	6.19669E-07	type3	Pathogenic/Likely pathogenic	Pathogenic
c.4904A>T	p.Asn1635Ile	rs778661133	missense	1.54907E-05	type2A	not provided	Uncertain significance
c.4888G>A	p.Val1630Met	NA	missense	6.19599E-07	type2A	not provided	Likely pathogenic
c.4883T>C	p.Ile1628Thr	rs61750584	missense	1.85891E-06	type2A	Pathogenic	Pathogenic
c.4841A>G	p.Asp1614Gly	rs61750582	missense	1.23924E-06	type2A	Uncertain significance	Likely pathogenic
c.4840G>A	p.Asp1614Asn	rs1478977315	missense	1.23913E-06	type2A	Uncertain significance	Likely pathogenic
c.4825G>A	p.Gly1609Arg	rs61750580	missense	3.09764E-06	type2A	Conflicting classifications of pathogenicity	Likely pathogenic
c.4789C>T	p.Arg1597Trp	rs61750117	missense	6.19568E-07	type2A	Pathogenic	Pathogenic
c.4752C>A	p.Tyr1584Ter	rs1475440343	stop gained	6.19636E-07	type3	Pathogenic	Pathogenic
c.4751A>G	p.Tyr1584Cys	rs1800386	missense	0.003442446	type1	Conflicting classifications of pathogenicity; risk factor	Uncertain significance
c.4747C>T	p.Arg1583Trp	rs61750116	missense	8.17968E-05	type1	Uncertain significance	Uncertain significance
c.4717G>A	p.Gly1573Ser	rs267607349	missense	1.11552E-05	type2A	Likely pathogenic	Likely pathogenic
c.4696C>T	p.Arg1566Ter	rs61750112	stop gained	4.33887E-06	type1	not provided	Likely pathogenic
c.4690C>T	p.Arg1564Trp	rs370854023	missense	6.8182E-06	type1	Conflicting classifications of pathogenicity	Uncertain significance
c.4580G>A	p.Arg1527Gln	rs780538558	missense	2.85042E-05	type2A	Conflicting classifications of pathogenicity	Uncertain significance
c.4570del	p.Val1524Ter	rs61750103	frameshift	6.19755E-07	type3	Pathogenic	Pathogenic
c.4552A>G	p.Lys1518Glu	rs61750102	missense	6.19612E-07	type2A	not provided	Likely pathogenic
c.4508T>A	p.Leu1503Gln	rs61750097	missense	6.21394E-07	type2A	Likely pathogenic	Pathogenic
c.4492G>A	p.Asp1498Asn	rs1009808532	missense	1.17731E-05	type2A	Uncertain significance	Uncertain significance
c.4430C>T	p.Thr1477Ile	NA	missense	6.19602E-07	type1	not provided	Likely pathogenic
c.4423C>T	p.Gln1475Ter	rs61750094	stop gained	6.19605E-07	type1	not provided	Pathogenic
c.4414 4415insC	p.Asp1472AlafsTer40	rs267607339	frameshift	6.19716E-07	type3	not provided	Pathogenic
c.4382C>T	p.Ala1461Val	rs61750089	missense	6.19623E-07	type2B	Pathogenic	Likely pathogenic
c.4378C>T	p.Leu1460Phe	rs61750088	missense	7.31132E-05	type2B	Likely pathogenic	Uncertain significance
c.4309G>A	p.Ala1437Thr	rs61750084	missense	3.71802E-06	type2M	Likely pathogenic	Pathogenic
c.4276C>T	p.Arg1426Cys	rs555366738	missense	6.19614E-06	type2M	not provided	Uncertain significance
c.4273A>T	p.Ile1425Phe	rs61750083	missense	6.19626E-07	type2M	Likely pathogenic	Pathogenic
c.4255C>A	p.His1419Asn	rs375498783	missense	3.71797E-05	type2A	Uncertain significance	Uncertain significance
c.4249G>T	p.Gly1417Trp	rs2136412846	missense	6.19593E-07	type2M	Likely pathogenic	Pathogenic
c.4247T>C	p.Ile1416Thr	rs61750081	missense	4.3374E-06	type2M	Pathogenic/Likely pathogenic	Pathogenic
c.4247T>A	p.Ile1416Asn	rs61750081	missense	1.85889E-06	type2A	Likely pathogenic	Pathogenic

c.4238C>T	p.Pro1413Leu	rs61750079	missense	1.36325E-05	type1	Uncertain significance	Uncertain significance
c.4205A>C	p.Gln1402Pro	NA	missense	6.19611E-07	type2M	Likely pathogenic	Likely pathogenic
c.4195C>T	p.Arg1399Cys	rs61750077	missense	5.14322E-05	type2M	Likely pathogenic	Likely pathogenic
c.4183C>T	p.Arg1395Trp	rs751394243	missense	1.23932E-05	type2M	Uncertain significance	Uncertain significance
c.4181C>T	p.Ser1394Phe	rs1033516182	missense	5.57682E-06	type2M	not provided	Uncertain significance
c.4162C>T	p.Gln1388Ter	rs1205530944	stop gained	6.19622E-07	type1	not provided	Pathogenic
c.4135C>T	p.Arg1379Cys	rs61750074	missense	2.54041E-05	type2B	Pathogenic/Likely pathogenic	Pathogenic
c.4133C>T	p.Ser1378Phe	rs61750073	missense	4.3373E-06	type1	Uncertain significance	Likely pathogenic
c.4121G>A	p.Arg1374His	rs61750072	missense	1.23925E-06	type2M	Pathogenic	Pathogenic
c.4120C>T	p.Arg1374Cys	rs61750071	missense	3.09829E-06	type2M	Pathogenic	Pathogenic
c.4117G>T	p.Asp1373Tyr	rs1332206266	missense	1.23926E-06	type2M	not provided	Likely pathogenic
c.4115T>G	p.Ile1372Ser	rs61750070	missense	3.53198E-05	type2B	Conflicting classifications of pathogenicity	Uncertain significance
c.4105T>A	p.Phe1369Ile	rs61750069	missense	6.19684E-06	type2M	Pathogenic/Likely pathogenic	Pathogenic
c.4094T>G	p.Leu1365Arg	NA	missense	6.19609E-07	type2M	not provided	Likely pathogenic
c.4082T>C	p.Leu1361Ser	rs61749408	missense	1.85884E-06	type1	Likely pathogenic	Pathogenic
c.4079T>C	p.Val1360Ala	rs267607338	missense	1.1156E-05	type2M	Uncertain significance	Uncertain significance
c.4075G>A	p.Glu1359Lys	rs61749407	missense	4.95721E-06	type2M	Pathogenic	Pathogenic
c.4024C>T	p.Arg1342Cys	rs61749404	missense	5.7003E-05	type1	Uncertain significance	Uncertain significance
c.4022G>A	p.Arg1341Gln	rs61749403	missense	1.2392E-06	type2B	Pathogenic	Pathogenic
c.4021C>T	p.Arg1341Trp	rs61749402	missense	1.85892E-05	type2B	Pathogenic/Likely pathogenic	Pathogenic
c.4010C>T	p.Pro1337Leu	rs61749400	missense	4.95684E-06	type2B	Pathogenic/Likely pathogenic	Pathogenic
c.4006C>T	p.Arg1336Ter	rs1565832211	stop gained	6.81555E-06	type3	Pathogenic	Likely pathogenic
c.4001G>A	p.Arg1334Gln	rs775812331	missense	6.19642E-06	type2M	Uncertain significance	Uncertain significance
c.4000C>T	p.Arg1334Trp	rs746810319	missense	2.41665E-05	type2M	Uncertain significance	Uncertain significance
c.3987C>G	p.Ile1329Met	rs138413641	missense	2.97437E-05	type1	not provided	Uncertain significance
c.3974C>T	p.Ser1325Phe	rs538488005	missense	6.1961E-07	type2M	Likely pathogenic	Likely pathogenic
c.3970G>A	p.Gly1324Ser	rs61749398	missense	4.33773E-06	type2M	Pathogenic	Pathogenic
c.3944G>A	p.Arg1315His	rs61749396	missense	1.67307E-05	type1	Likely pathogenic	Likely pathogenic
c.3943C>T	p.Arg1315Cys	rs61749395	missense	6.1962E-07	type3	Pathogenic/Likely pathogenic	Pathogenic
c.3931C>T	p.Gln1311Ter	rs267607337	stop gained	2.04502E-05	type3	Pathogenic	Pathogenic
c.3923G>T	p.Arg1308Leu	rs61749388	missense	6.196E-07	type2B	Likely pathogenic	Pathogenic
c.3923G>A	p.Arg1308His	rs61749388	missense	1.67292E-05	type2A	Uncertain significance	Uncertain significance
c.3917G>T	p.Arg1306Leu	rs61749385	missense	6.19596E-07	type2B	Pathogenic	Pathogenic
c.3917G>A	p.Arg1306Gln	rs61749385	missense	1.23928E-06	type2B	Pathogenic	Pathogenic
c.3839 3849del	p.Phe1280TrpfsTer9	rs2136413727	frameshift	1.23928E-06	type3	Pathogenic	Pathogenic

c.3797C>T	p.Pro1266Leu	rs61749370	missense	0.000667761	type2B	Conflicting classifications of pathogenicity	Uncertain significance
c.3797C>A	p.Pro1266Gln	rs61749370	missense	0.000541879	type2M	Conflicting classifications of pathogenicity	Uncertain significance
c.3773A>G	p.Tyr1258Cys	rs781111573	missense	2.16893E-05	type2B	not provided	Uncertain significance
c.3701G>A	p.Cys1234Tyr	NA	missense	6.19769E-07	type2A	not provided	Likely pathogenic
c.3679T>C	p.Cys1227Arg	rs61749366	missense	6.20284E-07	type1	Likely pathogenic	Pathogenic
c.3675-1G>A	NA	rs746457842	splice	9.30847E-06	type3	Likely pathogenic	Likely pathogenic
c.3613C>T	p.Arg1205Cys	rs373787920	missense	9.29286E-06	type1	Pathogenic/Likely pathogenic	Pathogenic
c.3613C>A	p.Arg1205Ser	NA	missense	2.4781E-06	type1	Likely pathogenic	Pathogenic
c.3586T>C	p.Cys1196Arg	rs61749365	missense	1.23899E-06	type2A	not provided	Likely pathogenic
c.3583G>T	p.Asp1195Tyr	rs374591991	missense	1.36299E-05	type2A	not provided	Likely pathogenic
c.3569G>A	p.Cys1190Tyr	rs1591865026	missense	6.19497E-07	type2A	Conflicting classifications of pathogenicity	Pathogenic
c.3467C>T	p.Thr1156Met	rs267607328	missense	7.43481E-06	type1	Pathogenic/Likely pathogenic	Pathogenic
c.3445T>C	p.Cys1149Arg	rs61748511	missense	1.84598E-06	type1	Pathogenic	Pathogenic
c.3437A>G	p.Tyr1146Cys	rs267607326	missense	1.83987E-06	type2A	Pathogenic	Pathogenic
c.3390C>T	p.Cys1130Cys	rs1591865617	synonymous	6.02112E-06	type1	Pathogenic/Likely pathogenic	Likely pathogenic
c.3379+1G>A	NA	rs2363337	splice	2.78866E-05	type3	Pathogenic	Pathogenic
c.3360G>A	p.Trp1120Ter	rs2136418333	stop gained	6.19618E-07	type3	Pathogenic	Pathogenic
c.3359G>C	p.Trp1120Ser	rs267607321	missense	1.23924E-06	type2A	Likely pathogenic	Pathogenic
c.3337C>T	p.Gln1113Ter	rs1944157065	stop gained	6.19656E-07	type3	not provided	Pathogenic
c.3332G>A	p.Cys1111Tyr	rs267607320	missense	6.19609E-07	type1	not provided	Likely pathogenic
c.3320A>G	p.Tyr1107Cys	rs267607319	missense	6.19645E-07	type2A	Uncertain significance	Likely pathogenic
c.3303C>A	p.Cys1101Ter	NA	stop gained	6.19626E-07	type3	not provided	Pathogenic
c.3271T>C	p.Cys1091Arg	NA	missense	6.19692E-07	type2A	not provided	Likely pathogenic
c.3232G>A	p.Glu1078Lys	rs267607316	missense	2.48081E-06	type2N	Likely pathogenic	Pathogenic
c.3212G>T	p.Cys1071Phe	rs267607315	missense	6.80835E-07	type3	not provided	Likely pathogenic
c.3159G>T	p.Gln1053His	rs61748496	missense	7.58653E-06	type2N	Likely pathogenic	Uncertain significance
c.3108+5G>A	NA	rs61748495	intron	1.85895E-06	type3	Conflicting classifications of pathogenicity	Uncertain significance
c.2967+1G>C	NA	NA	splice	6.1955E-07	type1	not provided	Uncertain significance
c.2944G>C	p.Val982Leu	rs376548659	missense	3.09783E-05	type3	not provided	Uncertain significance
c.2926C>T	p.Arg976Cys	rs764251476	missense	5.45265E-05	type2A	Uncertain significance	Uncertain significance
c.2900G>T	p.Gly967Val	rs141087261	missense	3.09764E-06	type3	not provided	Likely pathogenic
c.2879G>C	p.Arg960Pro	rs781033577	missense	4.95631E-06	type1	not provided	Uncertain significance
c.2878C>T	p.Arg960Trp	rs370984712	missense	0.000129488	type1	Uncertain significance	Uncertain significance
c.2820+5G>C	NA	rs569571599	intron	1.85852E-06	type3	not provided	Uncertain significance

c.2770C>T	p.Arg924Trp	rs61748491	missense	1.92062E-05	type1	Uncertain significance	Uncertain significance
c.2686G>T	p.Asp896Tyr	rs1022033438	missense	6.19592E-07	type3	Uncertain significance	Likely pathogenic
c.2686-1G>C	NA	rs61748488	splice	6.1954E-07	type1	Uncertain significance	Uncertain significance
c.2686-2A>G	NA	rs61748489	splice	6.19544E-07	type1	not provided	Uncertain significance
c.2635G>A	p.Asp879Asn	rs61748485	missense	9.91307E-06	type2N	Pathogenic	Pathogenic
c.2574C>G	p.Cys858Trp	rs184227165	missense	1.85846E-06	type1	not provided	Uncertain significance
c.2572T>A	p.Cys858Ser	rs1845232939	missense	6.19535E-07	type2N	not provided	Likely pathogenic
c.2561G>A	p.Arg854Gln	rs41276738	missense	0.004955972	type2N	Pathogenic	Pathogenic
c.2560C>T	p.Arg854Trp	rs61748482	missense	1.54887E-05	type2N	Likely pathogenic	Pathogenic
c.2554C>T	p.Gln852Ter	rs772534075	stop gained	1.61089E-05	type3	not provided	Likely pathogenic
c.2546G>T	p.Cys849Phe	rs772796741	missense	3.09761E-06	type2N	Likely pathogenic	Pathogenic
c.2516del	p.Gly839GlufsTer4	rs61748481	frameshift	4.33679E-05	type1	Likely pathogenic	Likely pathogenic
c.2447G>A	p.Arg816Gln	rs62643634	missense	1.30113E-05	type2N	Likely pathogenic	Pathogenic
c.2446C>T	p.Arg816Trp	rs121964894	missense	1.98274E-05	type2N	Pathogenic	Pathogenic
c.2443-1G>C	NA	rs61748480	splice	2.47812E-06	type3	Pathogenic/Likely pathogenic	Pathogenic
c.2442+4A>G	NA	rs777608246	intron	4.02742E-05	type1	Uncertain significance	Uncertain significance
c.2435del	p.Pro812ArgfsTer31	rs62643632	frameshift	7.18749E-05	type3	Pathogenic	Pathogenic
c.2435C>T	p.Pro812Leu	rs62643631	missense	4.58561E-05	type2N	Uncertain significance	Uncertain significance
c.2432C>T	p.Pro811Leu	rs1478975030	missense	1.23918E-06	type2N	not provided	Likely pathogenic
c.2430C>A	p.Cys810Ter	rs746648486	stop gained	6.19543E-07	type3	Pathogenic	Pathogenic
c.2396G>T	p.Cys799Phe	NA	missense	6.19499E-07	type2N	not provided	Likely pathogenic
c.2396G>A	p.Cys799Tyr	rs954273691	missense	1.85863E-06	type2N	Uncertain significance	Likely pathogenic
c.2390T>G	p.Leu797Arg	rs1219013109	missense	6.19521E-07	type2N	not provided	Likely pathogenic
c.2384A>G	p.Tyr795Cys	rs61748478	missense	1.23901E-06	type2N	Pathogenic	Likely pathogenic
c.2372C>T	p.Thr791Met	rs61748477	missense	1.79674E-05	type2N	Pathogenic	Pathogenic
c.2363G>A	p.Cys788Tyr	rs61748476	missense	6.19514E-07	type2N	Pathogenic	Pathogenic
c.2359G>A	p.Glu787Lys	rs61748474	missense	3.71709E-06	type2N	not provided	Uncertain significance
c.2345G>A	p.Arg782Gln	rs61748472	missense	1.05325E-05	type1	not provided	Uncertain significance
c.2344C>T	p.Arg782Trp	rs61748471	missense	3.40753E-05	type2N	Uncertain significance	Uncertain significance
c.2311A>G	p.Met771Val	rs1212894308	missense	5.57561E-06	type1	Uncertain significance	Uncertain significance
c.2303G>A	p.Arg768Gln	rs772203447	missense	2.29243E-05	type1	Likely pathogenic	Uncertain significance
c.2284A>G	p.Lys762Glu	rs267607310	missense	1.23909E-06	type1	not provided	Uncertain significance
c.2281+4A>G	NA	NA	intron	1.85873E-06	type1	not provided	Uncertain significance
c.2279G>A	p.Arg760His	rs61748467	missense	1.61102E-05	type2A	Pathogenic	Uncertain significance
c.2278C>T	p.Arg760Cys	rs61748466	missense	6.19559E-06	type2N	Conflicting classifications of pathogenicity	Uncertain significance

c.2269 2270del	p.Leu757ValfsTer22	rs61748465	frameshift	1.8587E-06	type3	Likely pathogenic	Pathogenic
c.2072del	p.Pro691GlnfsTer50	rs267607309	frameshift	2.47819E-06	type3	Pathogenic	Pathogenic
c.2067C>A	p.Cys689Ter	rs764755360	stop gained	2.47792E-06	type3	Pathogenic	Pathogenic
c.2016 2019del	p.Ser673ThrfsTer67	rs61748462	frameshift	1.23916E-06	type3	not provided	Pathogenic
c.1992del	p.Cys665AlafsTer3	rs1944531613	frameshift	6.19531E-07	type3	Pathogenic	Pathogenic
c.1931 1945+5del	NA	rs2136454053	splice	7.14818E-07	type3	Likely pathogenic	Uncertain significance
c.1930G>T	p.Glu644Ter	rs61748460	stop gained	2.72091E-06	type3	Pathogenic	Pathogenic
c.1926G>A	p.Trp642Ter	rs61754666	stop gained	6.77476E-07	type3	not provided	Pathogenic
c.1900G>C	p.Ala634Pro	rs1187357286	missense	1.31326E-06	type3	not provided	Likely pathogenic
c.1898G>A	p.Cys633Tyr	NA	missense	6.56413E-07	type1	not provided	Likely pathogenic
c.1897T>C	p.Cys633Arg	NA	missense	6.56415E-07	type3	not provided	Likely pathogenic
c.1892C>T	p.Ala631Val	rs199963222	missense	0.000230504	type1	Uncertain significance	Uncertain significance
c.1869C>G	p.Cys623Trp	rs61754017	missense	4.55956E-06	type2A	not provided	Uncertain significance
c.1812C>G	p.Tyr604Ter	rs1357055451	stop gained	1.29363E-06	type3	not provided	Pathogenic
c.1781C>G	p.Ala594Gly	rs267607308	missense	0.00012385	type1	Uncertain significance	Uncertain significance
c.1762A>C	p.Thr588Pro	NA	missense	6.46524E-07	type2A	not provided	Uncertain significance
c.1730-10C>A	NA	rs760570393	intron	9.79552E-06	type3	not provided	Uncertain significance
c.1729+3A>G	NA	NA	intron	1.24781E-06	type3	not provided	Uncertain significance
c.1729+3A>C	NA	rs2136455692	intron	6.23907E-07	type3	Uncertain significance	Uncertain significance
c.1693C>T	p.Gln565Ter	rs750364485	stop gained	6.2043E-07	type3	Pathogenic	Pathogenic
c.1672G>T	p.Asp558Tyr	rs1444535183	missense	1.23997E-06	type1	not provided	Uncertain significance
c.1668del	p.His556GlnfsTer21	NA	frameshift	1.23971E-06	type3	not provided	Pathogenic
c.1659G>A	p.Trp553Ter	rs2136455808	stop gained	6.19705E-07	type3	Pathogenic	Pathogenic
c.1648G>A	p.Gly550Arg	rs61754011	missense	6.19698E-07	type2A	Pathogenic	Likely pathogenic
c.1607T>C	p.Leu536Pro	rs1591890769	missense	3.71841E-06	type2A	Likely pathogenic	Pathogenic
c.1600G>A	p.Asp534Asn	rs1651544859	missense	1.23943E-06	type1	Uncertain significance	Likely pathogenic
c.1534-3C>A	NA	rs61754009	splice	2.47997E-06	type1	Likely pathogenic	Uncertain significance
c.1533+1G>A	NA	rs1555198839	splice	6.20317E-07	type3	Uncertain significance	Pathogenic
c.1497G>C	p.Gln499His	rs774725519	missense	4.95844E-06	type1	Uncertain significance	Uncertain significance
c.1339C>T	p.Arg447Trp	rs372495746	missense	3.77934E-05	type3	Uncertain significance	Uncertain significance
c.1309 1326del	p.Asp437 Arg442del	rs267607305	inframe deletion	1.23904E-06	type2A	Pathogenic	Likely pathogenic
c.1309G>T	p.Asp437Tyr	rs375486035	missense	4.58496E-05	type3	not provided	Uncertain significance
c.1255C>T	p.Gln419Ter	rs201379649	stop gained	6.19513E-07	type3	not provided	Pathogenic
c.1209C>G	p.Tyr403Ter	NA	stop gained	6.19508E-07	type3	not provided	Pathogenic
c.1156+2T>C	NA	NA	splice	6.19536E-07	type1	not provided	Pathogenic
c.1135T>G	p.Cys379Gly	rs763461692	missense	2.47803E-06	type1	not provided	Uncertain significance

c.1117C>T	p.Arg373Ter	rs62643625	stop gained	8.0545E-06	type3	Pathogenic	Pathogenic
c.1110-1G>A	NA	rs61754005	splice	1.23905E-06	type3	not provided	Uncertain significance
c.1109G>A	p.Cys370Tyr	rs763827767	missense	1.2395E-06	type3	not provided	Likely pathogenic
c.1093C>T	p.Arg365Ter	rs61754003	stop gained	6.19563E-06	type3	Pathogenic	Likely pathogenic
c.970C>T	p.Arg324Ter	rs61754000	stop gained	1.54904E-05	type3	Pathogenic	Pathogenic
c.962G>A	p.Cys321Tyr	rs766016814	missense	7.12499E-05	type1	not provided	Uncertain significance
c.902A>G	p.Tyr301Cys	rs1944807275	missense	2.47847E-06	type3	Uncertain significance	Likely pathogenic
c.884G>A	p.Cys295Tyr	rs770781601	missense	1.85876E-06	type3	not provided	Likely pathogenic
c.878del	p.Pro293GlnfsTer164	NA	frameshift	3.09807E-06	type3	not provided	Likely pathogenic
c.875-3C>A	NA	rs773976969	splice	1.85889E-06	type3	not provided	Uncertain significance
c.874+2T>C	NA	rs1944829900	splice	1.85897E-06	type3	Uncertain significance	Pathogenic
c.871del	p.Cys291AlafsTer166	rs1565853817	frameshift	6.19579E-07	type3	not provided	Pathogenic
c.817C>T	p.Arg273Trp	rs61753997	missense	3.77924E-05	type3	Pathogenic	Pathogenic
c.813C>G	p.Tyr271Ter	rs750697933	stop gained	1.23911E-06	type3	not provided	Pathogenic
c.788 811del	p.Cys263 Glu270del	rs63749067	inframe deletion	6.19587E-07	type3	Likely pathogenic	Likely pathogenic
c.763 766del	p.Cys255SerfsTer201	rs2136474390	frameshift	6.19543E-07	type3	Pathogenic	Pathogenic
c.760 761del	p.Leu254ValfsTer2	rs1435596998	frameshift	2.47799E-06	type3	not provided	Pathogenic
c.722T>G	p.Val241Gly	rs2136474453	missense	6.19549E-07	type1	Uncertain significance	Uncertain significance
c.706C>T	p.Arg236Cys	rs140912382	missense	0.000223036	type1	Uncertain significance	Uncertain significance
c.658-3C>A	NA	rs377196768	splice	8.80456E-05	type3	Conflicting classifications of pathogenicity	Uncertain significance
c.605G>C	p.Arg202Pro	rs369737556	missense	5.57637E-06	type2A	not provided	Uncertain significance
c.605G>A	p.Arg202Gln	rs369737556	missense	1.05332E-05	type2A	Uncertain significance	Uncertain significance
c.604C>T	p.Arg202Trp	rs990682639	missense	1.85877E-05	type2A	Uncertain significance	Uncertain significance
c.592C>T	p.Gln198Ter	rs2136500386	stop gained	6.19523E-07	type3	Likely pathogenic	Pathogenic
c.533-3C>G	NA	rs2136500500	splice	6.19507E-07	type3	Uncertain significance	Uncertain significance
c.514G>A	p.Asp172Asn	rs766305860	missense	1.85856E-06	type2A	not provided	Likely pathogenic
c.497A>T	p.Asn166Ile	rs62643622	missense	6.19512E-07	type1	not provided	Likely pathogenic
c.493T>G	p.Phe165Val	rs754520488	missense	6.19503E-07	type3	not provided	Likely pathogenic
c.478G>A	p.Gly160Arg	NA	missense	1.23906E-06	type3	not provided	Pathogenic
c.469A>G	p.Lys157Glu	rs553810662	missense	1.239E-06	type3	not provided	Likely pathogenic
c.449T>C	p.Leu150Pro	rs61753994	missense	1.23898E-06	type3	Likely pathogenic	Pathogenic
c.440A>G	p.Gln147Arg	rs1483313796	missense	1.23917E-06	type1	not provided	Uncertain significance
c.414 426del	p.Arg139AlafsTer32	rs1312486904	frameshift	1.23902E-06	type3	Pathogenic	Pathogenic
c.421G>T	p.Asp141Tyr	rs61753992	missense	6.19513E-07	type3	not provided	Pathogenic
c.374 387del	p.Gly125ValfsTer3	rs63749066	frameshift	6.19505E-07	type3	Likely pathogenic	Pathogenic
c.311 312del	p.Gln104ArgfsTer19	rs1481396407	frameshift	4.33726E-06	type3	Pathogenic	Likely pathogenic
c.310C>T	p.Gln104Ter	rs2136522699	stop gained	6.19556E-07	type3	Pathogenic	Pathogenic

c.260A>C	p.Tyr87Ser	rs62643621	missense	1.48701E-05	type2A	not provided	Uncertain significance
c.257T>A	p.Val86Glu	NA	missense	1.23903E-06	type3	Likely pathogenic	Likely pathogenic
c.250C>T	p.Leu84Phe	rs372664002	missense	0.000104711	type2A	Uncertain significance	Uncertain significance
c.236G>T	p.Gly79Val	rs1020664699	missense	1.92074E-05	type3	Uncertain significance	Uncertain significance
c.191del	p.Gly64AlafsTer19	rs62643618	frameshift	6.19543E-07	type3	not provided	Pathogenic
c.164G>A	p.Gly55Glu	NA	missense	6.19493E-07	type2A	not provided	Pathogenic
c.115G>A	p.Gly39Arg	rs1397778191	missense	8.67373E-06	type1	Likely pathogenic	Likely pathogenic
c.103T>C	p.Cys35Arg	NA	missense	1.23898E-06	type3	not provided	Likely pathogenic
c.100C>T	p.Arg34Ter	rs61753984	stop gained	7.43398E-06	type3	Pathogenic	Pathogenic
c.100C>G	p.Arg34Gly	rs61753984	missense	6.19498E-07	type3	not provided	Likely pathogenic

Supplementary Table S2. Estimated global prevalence of autosomal dominant von Willebrand disease (VWD) type 1.

Population	Total number of alleles	Total number of affected alleles by Type 1 variants	Frequency of affected alleles	Prevalence per 10^3 individuals (95% CI)
**General**	1614324	8540	0.0053	10.6 (10.4–10.8)
**African/ AfricanAmerican**	75090	302	0.004	8.2 (7.23–9.1)
**Admixed American**	60038	204	0.0034	7.2 (6.3–8.2)
**Ashkenazi Jewish**	29608	109	0.0037	7.3 (6–8.7)
**East.Asian**	44896	99	0.0022	4.4 (3.55–5.3)
**European Finnish**	64052	265	0.0041	8.3 (7.3–9.2)
**Middle.Eastern**	6062	30	0.005	10.6 (6.9–14.4)
**European non Finnish**	1180062	7076	0.006	12 (11.7–12.2)
**South Asian**	91092	150	0.002	3.3 (2.8–3.8)
**Remaining**	64336	305	0.005	9.7 (8.6–10.8)

Supplementary Table S3. Estimated global prevalence of autosomal dominant von Willebrand disease (VWD) type 2A.

Population	Total number of alleles	Total number of affected alleles by Type 2A variants	Frequency of affected alleles	Prevalence per 10^3 individuals (95% CI)
**General**	1614324	1036	0.0006	1.3 (1.2–1.4)
**African/ AfricanAmerican**	75090	37	0.0005	1.0 (0.7–1.4)
**Admixed American**	60038	88	0.0015	2.9 (2.3–3.6)
**Ashkenazi Jewish**	29608	4	0.0001	0.3 (0.07–0.5)
**East.Asian**	44896	99	0.0022	4.4 (3.6–5.3)
**European Finnish**	64052	53	0.0008	1.7 (1.2–2.1)
**Middle.Eastern**	6062	7	0.0012	2.3 (0.7–4.3)
**European non Finnish**	1180062	632	0.0005	1.1 (1.0–1.2)
**South Asian**	91092	48	0.0005	1.1 (0.8–1.4)
**Remaining**	64336	68	0.0011	2.1 (1.6–2.7)

Supplementary Table S4. Estimated global prevalence of autosomal dominant von Willebrand disease (VWD) type 2B.

Population	Total number of alleles	Total number of affected alleles by Type 2B variants	Frequency of affected alleles	Prevalence per 10^3 individuals (95% CI)
**General**	1614324	1373	0.00085	1.7 (1.6–1.8)
**African/ AfricanAmerican**	75090	20	0.00027	0.5 (0.3–0.8)
**Admixed American**	60038	74	0.00123	2.5 (1.9–3.0)
**Ashkenazi Jewish**	29608	98	0.00331	6.6 (5.3–7.9)
**East.Asian**	44896	1	0.00002	0.04 (0–0.13)
**European Finnish**	64052	285	0.00445	8.9 (7.8–9.9)
**Middle.Eastern**	6062	3	0.00049	1.0 (0–2.3)
**European non Finnish**	1180062	789	0.00067	1.3 (1.2–1.4)
**South Asian**	91092	42	0.00046	0.9 (0.7–1.2)
**Remaining**	64336	61	0.00095	1.9 (1.4–2.4)

Supplementary Table S5. Estimated global prevalence of autosomal dominant von Willebrand disease (VWD) type 2M.

Population	Total number of alleles	Total number of affected alleles by Type 2M variants	Frequency of affected alleles	Prevalence per 10^3 individuals (95% CI)
**General**	1614324	1234	0.00077	1.5 (1.4–1.6)
**African/ AfricanAmerican**	75090	28	0.00037	0.7 (0.5–1.0)
**Admixed American**	60038	43	0.00072	1.4 (1.0–1.9)
**Ashkenazi Jewish**	29608	1	0.00007	0.1 (0–0.2)
**East.Asian**	44896	22	0.00049	1.0 (0.6–1.4)
**European Finnish**	64052	1	0.00002	0.03 (0–0.09)
**Middle.Eastern**	6062	9	0.00148	3.0 (1.3–4.9)
**European non Finnish**	1180062	615	0.00052	1.0 (1.0–1.1)
**South Asian**	91092	445	0.00489	9.7 (8.8–10.6)
**Remaining**	64336	70	0.00109	2.2 (1.7–2.7)

Supplementary Table S6. Estimated global prevalence of autosomal recessive von Willebrand disease (VWD) type 2N.

Population	Total number of alleles	Total number of affected alleles by types 1_3 null /type 2N variants	Frequency of affected alleles	Prevalence per 10^6 individuals (95% CI)
**General**	1614324	8950	0.00582	33.9 (32.5–35.2)
**African/ AfricanAmerican**	75090	139	0.00206	4.2 (3.0–5.7)
**Admixed American**	60038	194	0.00330	10.9 (8.1–14.2)
**Ashkenazi Jewish**	29608	42	0.00142	2.0 (1.0–3.4)
**East.Asian**	44896	10	0.00036	0.1 (0.04–0.29)
**European Finnish**	64052	421	0.00669	44.7 (36.7–53.6)
**Middle.Eastern**	6062	9	0.00148	2.2 (0.4–6.1)
**European non Finnish**	1180062	7637	0.00680	46.2 (44.2–48.2)
**South Asian**	91092	184	0.00210	4.4 (3.2–5.8)
**Remaining**	64336	314	0.00529	27.9 (22.4–34.2)

Supplementary Table S7. Estimated global prevalence of autosomal recessive von Willebrand disease (VWD) type 3.

Population	Total number of alleles	Total number of affected alleles by Type1_null and/or type 3 variants	Frequency of affected alleles	Prevalence per 10^6 individuals (95% CI)
**General**	1614324	1742	0.00116	1.3 (1.2–1.5)
**African/ AfricanAmerican**	75090	47	0.00063	0.4 (0.2–0.7)
**Admixed American**	60038	27	0.00045	0.2 (0.08–0.4)
**Ashkenazi Jewish**	29608	3	0.00010	0.01 (0–0.06)
**East.Asian**	44896	28	0.00067	0.4 (0.2–0.8)
**European Finnish**	64052	73	0.00117	1.3 (0.8–2.1)
**Middle.Eastern**	6062	5	0.00083	0.7 (0.03–2.7)
**European non Finnish**	1180062	1090	0.00102	1.0 (0.9–1.2)
**South Asian**	91092	401	0.00442	19.5 (16.0–23.6)
**Remaining**	64336	68	0.00116	1.3 (0.8–2.0)

Supplementary Table S8. Estimated global prevalence of autosomal recessive von Willebrand disease (VWD) type 3 including SVs and CNVs variants.

Population	Prevalence per 10^6 individuals (95% CI)
**General**	1.8 (1.66–1.96)
**African/ AfricanAmerican**	0.7 (0.42–1.1)
**Admixed American**	0.2 (0.08–0.4)
**Ashkenazi Jewish**	0.039 (0.005–0.14)
**East.Asian**	2 (1.14–3.1)
**European Finnish**	1.4 (0.9–2.06)
**Middle.Eastern**	0.7 (0.027–2.7)
**European non Finnish**	1.5 (1.307–1.6)
**South Asian**	19.8 (16.1–23.9)
**Remaining**	1.6 (1.02–2.4)

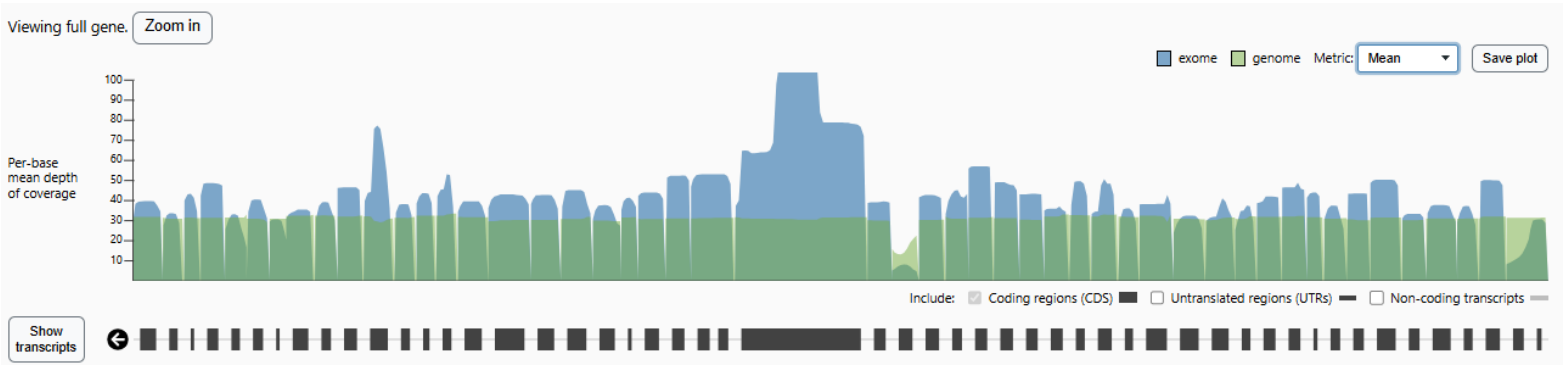

Figure S1. Mean depth of coverage per base of *VWF* for exome (blue) and genome (green) data of the gnomAD database (https://gnomad.broadinstitute.org/gene/ENSG00000110799?dataset=gnomad_r4), ENST00000261405.10 / NM_000552.5.
